# World Endometriosis Research Foundation Endometriosis Phenome and Biobanking Harmonization Project: III. Fluid biospecimen collection, processing, and storage in endometriosis research

**DOI:** 10.1016/j.fertnstert.2014.07.1208

**Published:** 2014-11

**Authors:** Nilufer Rahmioglu, Amelie Fassbender, Allison F. Vitonis, Shelley S. Tworoger, Lone Hummelshoj, Thomas M. D'Hooghe, G. David Adamson, Linda C. Giudice, Christian M. Becker, Krina T. Zondervan, Stacey A. Missmer, WERF EPHect Working Group, G.D. Adamson, C. Allaire, R. Anchan, C.M. Becker, M.A. Bedaiwy, G.M. Buck Louis, C. Calhaz-Jorge, K. Chwalisz, T.M. D'Hooghe, A. Fassbender, T. Faustmann, A.T. Fazleabas, I. Flores, A. Forman, I. Fraser, L.C. Giudice, M. Gotte, P. Gregersen, S.-W. Guo, T. Harada, D. Hartwell, A.W. Horne, M.L. Hull, L. Hummelshoj, M.G. Ibrahim, L. Kiesel, M.R. Laufer, K. Machens, S. Mechsner, S.A. Missmer, G.W. Montgomery, A. Nap, M. Nyegaard, K.G. Osteen, C.A. Petta, N. Rahmioglu, S.P. Renner, J. Riedlinger, S. Roehrich, P.A. Rogers, L. Rombauts, A. Salumets, E. Saridogan, T. Seckin, P. Stratton, K.L. Sharpe-Timms, S. Tworoger, P. Vigano, K. Vincent, A.F. Vitonis, U.-H. Wienhues-Thelen, P.P. Yeung, P. Yong, K.T. Zondervan

**Affiliations:** a Wellcome Trust Centre for Human Genetics, University of Oxford, Oxford, United Kingdom; b Organ Systems, Department of Development and Regeneration, Katholieke Universiteit Leuven, Leuven, Belgium; c Department of Obstetrics and Gynaecology, Leuven University Fertility Centre, University Hospital Leuven, Leuven, Belgium; d Department of Obstetrics, Gynecology, and Reproductive Biology, Brigham and Women's Hospital and Harvard Medical School, Boston, Massachusetts; e Boston Center for Endometriosis, Boston Children's Hospital and Brigham & Women's Hospital, Boston, Massachusetts; f Channing Division of Network Medicine, Department of Medicine, Brigham and Women's Hospital and Harvard Medical School, Boston, Massachusetts; g Department of Epidemiology, Harvard School of Public Health, Boston, Massachusetts; h World Endometriosis Research Foundation (WERF), London, United Kingdom; i Palo Alto Medical Foundation Fertility Physicians of Northern California, Palo Alto, California; j University of California San Francisco, San Francisco, California; k Nuffield Department of Obstetrics & Gynaecology, University of Oxford, Oxford, United Kingdom; l Endometriosis CaRe Centre Oxford, University of Oxford, Oxford, United Kingdom

**Keywords:** Endometriosis, standardization, standard operating procedures, biological fluid samples, EPHect

## Abstract

**Objective:**

To harmonize standard operating procedures (SOPs) and standardize the recording of associated data for collection, processing, and storage of fluid biospecimens relevant to endometriosis.

**Design:**

An international collaboration involving 34 clinical/academic centers and 3 industry collaborators from 16 countries on 5 continents.

**Setting:**

In 2013, 2 workshops were conducted, followed by global consultation, bringing together 54 leaders in endometriosis research and sample processing worldwide.

**Patient(s):**

None.

**Intervention(s):**

Consensus SOPs were based on: [1] systematic comparison of SOPs from 18 global centers collecting fluid samples from women with and without endometriosis on a medium/large scale (publication on >100 cases), [2] literature evidence where available, or consultation with laboratory experts otherwise, and [3] several global consultation rounds.

**Main Outcome Measure(s):**

Standard recommended and minimum required SOPs for biofluid collection, processing, and storage in endometriosis research.

**Result(s):**

We developed recommended standard and minimum required SOPs for the collection, processing, and storage of plasma, serum, saliva, urine, endometrial/peritoneal fluid, and menstrual effluent, and a biospecimen data-collection form necessary for interpretation of sample-derived results.

**Conclusion(s):**

The Endometriosis Phenome and Biobanking Harmonisation Project SOPs allow endometriosis research centers to decrease variability in biofluid sample results, facilitating between-center comparisons and collaborations. The procedures are also relevant to research into other female conditions involving biofluid samples subject to cyclic reproductive influences. The consensus SOPs are based on the best available evidence; areas with limited evidence are identified as requiring further pilot studies. The SOPs will be reviewed based on investigator feedback, and through systematic tri-annual follow-up. Updated versions will be made available at: endometriosisfoundation.org/ephect.

**Discuss:** You can discuss this article with its authors and with other ASRM members at **http://fertstertforum.com/rahmioglun-werf-ephect-iii/**

Many centers worldwide have been collecting blood and other fluid samples from women with and without endometriosis, with the aim of identifying potential diagnostic biomarkers and novel drug targets for the disease (1). Molecular profiles obtained toward these goals include, but are not limited to, changes at the deoxyribonucleic acid (DNA), ribonucleic acid (RNA), protein, and metabolite levels detected in various bodily fluids. However, variability in specimen collection, processing, and storage methods can act as a considerable source of bias and measurement error, obscuring or entirely preventing detection of disease-related molecular perturbations 2, 3.

Standard operating procedures (SOPs) and recommendations for blood collection in reproductive biology research have been published 4, 5, but none of these exist for other fluid specimens such as urine, saliva, or peritoneal and endometrial fluid. The majority of biospecimens collected for endometriosis research worldwide are collected and processed using different, sometimes nonspecified SOPs, making comparisons among studies, and data pooling, extremely difficult. Standardized collection of biospecimens across centers using internationally agreed-on SOPs—based on existing scientific evidence and consensus—is likely to reduce variability and facilitate comparability of results and enhance the detection of endometriosis biomarker relationships through multi-center collaborative studies. It would also allow meaningful comparison among different patient subpopulations and ethnic groups, and enable adequately powered, targeted studies on such groups that are less prone to between-center technical variability in results. Successful investigation of fluid markers among many centers, in both formally designed and ad hoc consortia utilizing de novo sample collection or pooled existing data, has been well established in the investigation of other disease outcomes 6, 7, 8, 9, 10, 11, 12.

Efforts to create and share evidence-based SOPs are underway in several research fields, such as those led by the National Cancer Institute (NCI) in the United States 13, 14, the Biobanking and Biomolecular Resource Research Infrastructure (BBMRI) program in Europe (15), and many other international organizations, for various purposes (Table 1). A successful model for the impact that standardized biobanking can have on research is British Columbia's multi-institutional and multidisciplinary ovarian cancer research group (OVCARE). Founded in 2000, this initiative has grown from a small group of researchers and disconnected research projects to a coherent team that is recognized internationally as leading the study of ovarian cancer, exemplified by a series of important biobank-based publications that have fundamentally changed the way ovarian cancer research is being performed 16, 17, 18.

**Table 1:** List of major organizations that have published best-practice documents regarding biospecimen collections for research or clinical use.

Organization	URL
National Cancer Institute: Biorepositories and Biospecimen Research Branch	http://biospecimens.cancer.gov/practices/
International Society for Biological and Environmental Biorepositories (ISBER)	http://c.ymcdn.com/sites/www.isber.org/resource/resmgr/Files/2012ISBERBestPractices3rdedi.pdf
The International Agency for Research on Cancer (IARC)	http://ibb.iarc.fr/docs/recommendations_BRC.pdf
The Organisation for Economic Cooperation and Development (OECD)	http://www.oecd.org/dataoecd/7/13/38777417.pdf
Medical Research Council (MRC)	http://www.mrc.ac.uk/news-events/publications/human-tissue-and-biological-samples-for-use-in-research/
Australian Biospecimen Network (ABN)	http://abrn.net/what-we-do/protocols/
American Society of Clinical Pathology (ASCP)	http://www.ascp.org/PDF/BOC-PDFs/CMP/CMPBooklet.pdf

The mission of the World Endometriosis Research Foundation (WERF) Endometriosis Phenome and Biobanking Harmonisation Project (EPHect) is to develop a consensus on standardization and harmonization of phenotypic surgical/clinical data and biologic sample–collection methods in endometriosis research. Specifically, EPHect provides evidence-based guidelines to facilitate large-scale, internationally collaborative, longitudinal, epidemiologically robust, translational biomarker and treatment target–discovery research in endometriosis. The guidelines are on: [1] detailed surgical and clinical and epidemiologic phenotyping (phenome) data to be collected from women with and without endometriosis to allow collaborative subphenotype discovery and validation analyses; and [2] SOPs for collection, processing, and long-term storage of biologic samples from women with and without endometriosis. To the best of our knowledge, this harmonization initiative is unique in terms of its scope, as it addresses standardization of phenotypic data collection and biologic sampling procedures simultaneously for a specific disease, based on consensus from a large group of academic and industrial leaders in endometriosis research. The initiative is a direct response to the key priorities of phenome data collection and SOP harmonization identified in Endometriosis Research Directions workshops held in 2008 (19) and 2011 (1), and it will allow the investigation of a substantial number of other research priorities highlighted.

The present article describes the development of evidence-based SOPs for the collection, processing, and storage of 6 fluid-sample types relevant to endometriosis research: blood, urine, saliva, peritoneal fluid, endometrial fluid, and menstrual fluid. The development of the surgical (EPHect SSF and MSF) (20) and clinical questionnaires (EPHect EPQ-S and EPQ-M) (21) for standardized phenotypic data collection were described in our 2 previous articles in the series; evidence-based SOPs for tissue (ectopic and eutopic endometrium, myometrium, and peritoneum) collection are described in our final article (22).

## Methods

**Figure 1:**
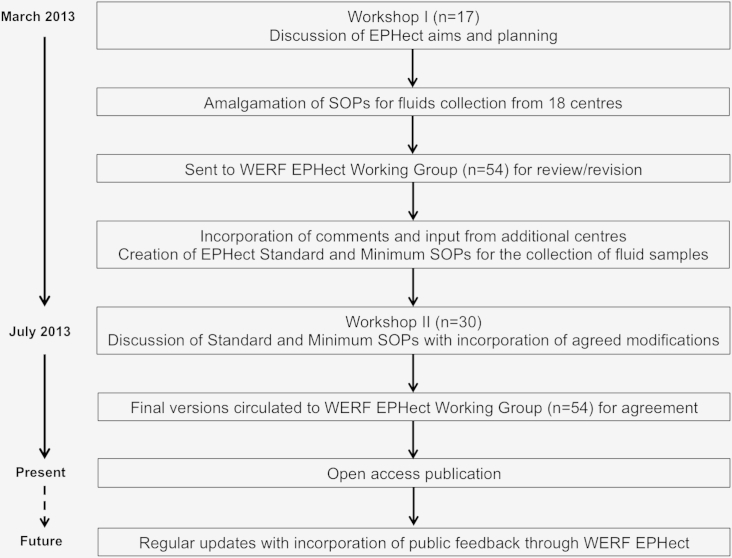
Flow diagram depicting the WERF EPHect development and consensus process (biological fluid sample SOPs).

We conducted 2 workshops in March and July 2013, followed by several rounds of expert review, bringing together 54 leaders in endometriosis research and management and in sample processing from 34 clinical/academic centers and 3 industry collaborators in 16 countries to develop and reach consensus on evidence-based phenome collection and SOP guidelines (Fig. 1; 20). During workshop I and a subsequent consultation round, we identified 18 centers worldwide that collect biologic fluid samples from endometriosis cases and controls on a large scale (criterion: publication on >100 cases); all provided SOPs for sample collection, processing, and storage. Six fluid sample types were collected by the centers (blood, urine, saliva, peritoneal fluid, endometrial fluid, and menstrual fluid; Table 2). Quantities collected were often a balance between volumes sufficient to conduct a wide range of future experiments (biobanking); participant-based acceptability; and the costs of collection, processing, and long-term storage.

**Table 2:** Number of centers, and the ranges of quantities collected for blood, urine, saliva, peritoneal fluid, endometrial fluid, and menstrual effluent.

	No. of centers	Quantities collected
Blood	18	10–35 ml
Urine	6	20–120 ml
Saliva	4	500 μl–2 ml
Peritoneal fluid	8	1–25 ml
Endometrial fluid	6	20–400 μl
Menstrual effluent	3	No data

In addition to the information provided by the 18 centers, we searched for publicly available SOPs from general large-scale biobanking efforts (e.g., UK Biobank); large biorepositories (International Society for Biological and Environmental Biorepositories [ISBER]; the NCI Biorepositories and Biospecimen Research Branch [NCI-BBRB]; and the Australian Biospecimen Network [ABRN]). A systematic literature search was conducted in PubMed for English-language publications describing (crucial steps in) SOPs, using the following search terms: “standard operating procedure” with “endometriosis” or “blood” or “urine” or “endometrial fluid” or “peritoneal fluid” or “menstrual effluent” or “fluid samples” or “best practice” or “biobank.” Reference lists of retrieved articles were hand-searched for additional references and material. In addition, online material from biobanks and biorepositories was sought through the Google search engine with the same search terms. On the basis of this information, we compiled draft consensus SOPs, identifying steps that varied between center-specific SOPs, but for which little or no evidence could be obtained. Prior to workshop II, consensus documents and associated evidence and queries were distributed to the WERF EPHect Working Group. During workshop II, and a separate e-mail consultation process among those who were unable to attend the workshop, the final consensus SOPs were reviewed and agreed on (Supplemental Tables 1–6 and Appendixes 1–6, available online).

Although validity, reliability, and scientific advancement are the main goals of EPHect, an important point acknowledged by the WERF EPHect Working Group was that there are likely to be differences in resources and logistics among centers that may mean they are unable to adhere to some of the strictest protocol standards. All experts therefore agreed on 2 tiers for most steps in the SOPs: standard recommended and minimum required. We strongly advise standard recommended collection SOPs to be adopted when possible, as they will yield results that are least prone to variation and degradation of the samples; the minimum required SOP steps are offered to provide the fundamentals for standardization that need to be adhered to as an absolute minimum requirement given unavoidable logistical and budgetary circumstances. It is important to note that publications of results generated using samples collected following the WERF EPHect SOPs need to state explicitly which EPHect procedures were used and any alterations made to them. Following good scientific practice, we strongly recommend that each center maintain a copy of the details of the exact protocol used.

When collecting biologic samples for research purposes, additional data items need to be collected to allow interpretation of results from the samples, such as recent medication use by the participant and her menstrual-cycle phase at the time of sample collection. For this purpose, the WERF EPHect Working Group developed a consensus Biospecimen Form (Supplemental Appendix 7, available online) to be completed at each sample collection event.

Approval by an ethics committee or institutional review board was not required for formation of the WERF EPHect Working Group, review of existing literature, nor consensus regarding best practices for endometriosis research described within the WERF EPHect 4-article series. This endeavor did not include data from human subjects. A comprehensive list of declared conflicts of interest for each of the authors and members of the WERF EPHect Working Group is provided.

## Results

Below, we describe the rationale behind the development of the WERF EPHect SOPs for the collection, processing, and storage of blood and its derivatives (serum, plasma, and red/white blood cells), urine, saliva, peritoneal fluid, endometrial fluid, and menstrual effluent.

### Blood

**Figure 2:**
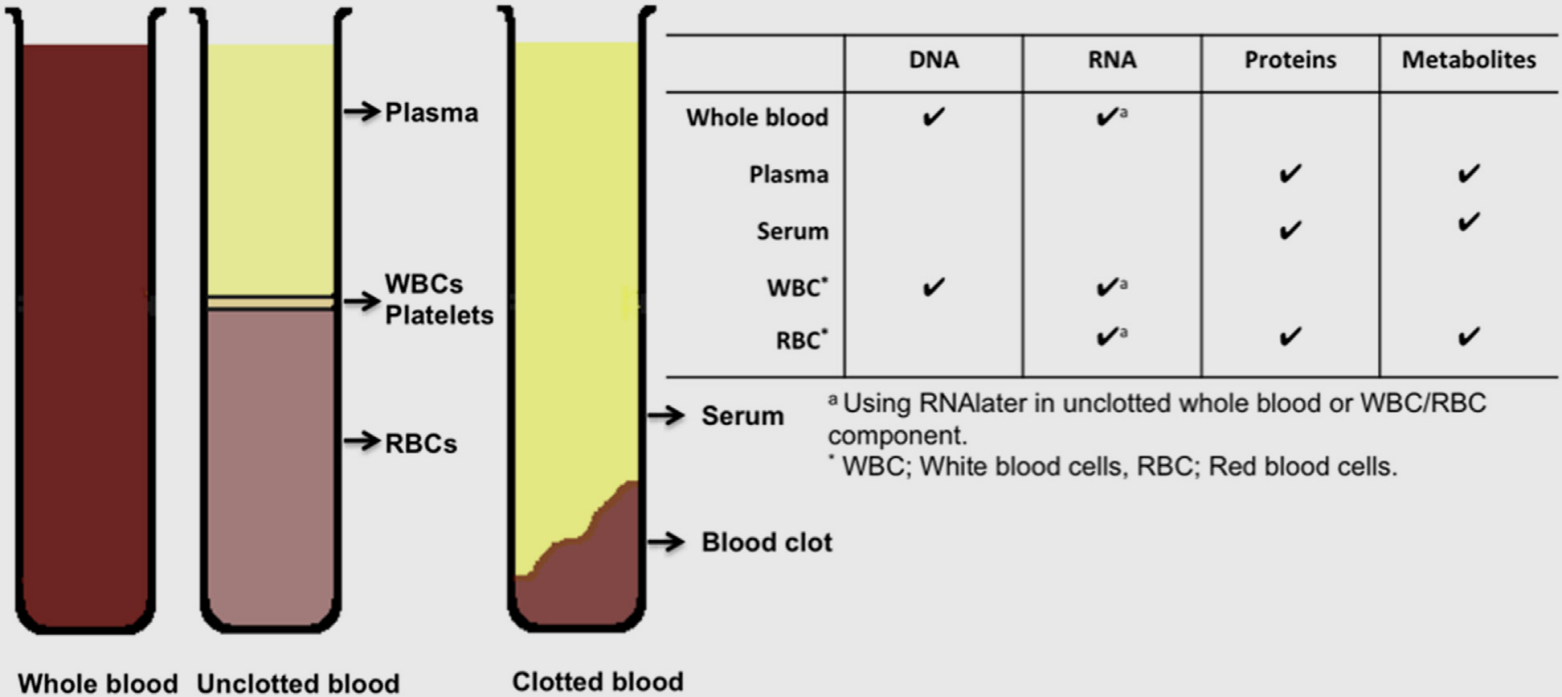
Potential uses of blood constituents in genetic, expression, protein, and metabolite analyses.

Blood is most usefully banked after separation into its derivatives (serum, plasma, and red/white blood cells), to allow the widest possible future use (Fig. 2). Peripheral blood allows the measurement of a broad range of biomolecules, in both patients and healthy volunteers, and relatively large volumes can be collected. However, peripheral blood includes a complex mix of molecules reflecting many biologic processes in the body, in which biologic changes relevant to the disease may not be detected as readily as in a disease-related tissue. When collecting blood samples for a study, several important decisions need to be made regarding: [1] timing and conditions of sample collection; [2] use of anticoagulants and clot accelerators in collection tubes; [3] sample stability between collection and processing; [4] processing; and [5] long-term storage. We describe important aspects of each of these, and how they are dealt with in the SOPs. Eighteen EPHect centers provided blood SOPs for consensus agreement.

#### Timing and conditions of sample collection

The time of day that blood is collected from a participant is crucial if the aim is to measure biomarkers that are affected by physiological state, circadian rhythms, fasting status, or other factors that could result in changes in the endogenous concentrations of these biomolecules. For example, many metabolic markers, as well as certain hormones such as insulin, change in concentration after food consumption. Therefore, the time since the participant had anything to eat or drink except plain water should be recorded, and ideally samples should be collected after a 10-hour fast (23). If blood samples are collected on the day of surgery, they should be collected prior to induction of anesthesia, as anesthetic drugs can have a profound effect on biomarker detection (24).

It is important to consider any other aspects of the timing of sample collection that could bias measurements. Recording the date of sample collection is universally important, particularly if biomarkers with seasonal variation (e.g., vitamin D) are of interest. Menstrual details including the last menstrual period (LMP) date should be recorded, to allow for menstrual cycle variation in analysis. Recent drugs used should be recorded. The WERF EPHect SOPs and biospecimen data collection form take account of these important considerations; however, if the investigator has a specific interest in a particular biomarker, it is advisable to test the sensitivity of its measurement to the specified sampling timing and conditions. An example would be the measurement of stress hormones (cortisol), which will be heavily influenced by not only the diurnal cycles but also the physiological and psychological state of the participant and time of day (25) and therefore should not be conducted on the day of surgery.

#### Anticoagulants and clot accelerators

The type of anticoagulant used in tubes for blood sample collection affects how the sample can be used (26). Detected concentration levels of certain biomarkers can vary with the type of anticoagulant used. For example, it has been shown that concentration of tumor necrosis factor–alpha and interleukin-6 are highest in ethylenediaminetetraacetic acid (EDTA) plasma in comparison to heparin and citrate plasma samples (27), which are the most commonly used types of anticoagulants. Particular anticoagulants are recommended, or even required, for certain analytical purposes (28). EDTA tubes are often the first preferred type, as they are suitable for a wide range of DNA-based and protein assays (26). Lithium–heparin is preferred for plasma-based metabolomic studies (26). Citrate tubes are recommended if the interest is to perform functional clotting-factor assays (26); however, this tube type is not commonly a priority as the liquid anticoagulant leads to lower biomarker concentrations as a result of dilution, dependent on the individual's hematocrit and sample volume (29).

To obtain serum samples, whole blood needs to be clotted and the supernatant (serum) removed. Clots form very slowly in tubes left untreated, and serum separator tubes with clot accelerators (most notably silica and thrombin) are available to speed up the process. Silica is most commonly used because of relatively low cost and because it does not appear to affect measurable concentrations for a range of different assays 30, 31, 32, 33. Serum samples are suitable for most clinical biochemistry and metabolomic studies, but they may not be optimal for other assays, such as proteomics, because clot-related peptides can contaminate the sample 34, 35. As an illustration of biobank-based collection tube prioritization, UK Biobank collects 45 ml of blood from each participant and prioritizes EDTA and lithium–heparin plasma tubes, as they apply to a wide range of DNA-based and protein assays. For serum collection, they use silica as a clotting factor (26).

#### Sample stability between collection and processing/storage

The time lapse between sample collection and processing/storage, as well as temperature conditions, are crucial variables affecting the stability of molecules in samples. In general, keeping samples at 4°C (or on ice) from collection until storage minimizes enzymatic degradation of many biomolecules (36). The length of time for which biomolecules are stable is variable. DNA is one of the most stable biomolecules (36); certain metabolites begin to degrade within 2 hours after blood sample collection (37), whereas substantial degradation of messenger ribonucleic acid (mRNA) occurs within the first half hour (4).

For most uses, therefore, blood samples should be processed and stored as soon as possible (within 2 hours) or at most within 4 hours 37, 38. If there is a longer delay in processing, pilot studies should be conducted to test the stability of individual biomarkers, as some biomarkers are stable for up to 48 hours 39, 40. Such pilot studies to test measurement sensitivity are recommended as standard in any case where the biomolecule of interest is defined from the outset. RNA integrity is maintained with the immediate postcollection addition of commercially available inhibitors of RNAse enzymes such as RNAlater^®^ or PAXgene tubes^®^, although these can be costly on a large scale (26). RNAse inhibitors compromise the utility of the samples for other assays, and therefore a separate blood sample aliquot specific for RNA analysis should be collected if possible within study budget constraints. Live cells harvested for direct experiments or culture are stable at room temperature for up to 48 hours, but within that time window, they should be either cultured or cryopreserved in liquid nitrogen (LN_2_) after pretreatment with dimethylsulfoxide (DMSO) to avoid cell rupture 26, 41.

#### Processing

Centrifugation is performed to separate blood into its constituent components. The duration, speed, and temperature conditions under which centrifugation is performed vary considerably among centers. For example, the UK Biobank centrifuges blood samples at 2,500 x g for 10 minutes at 4°C (26), and the NCI recommends centrifuging 1,100–1,300 x g for 10–20 minutes at room temperature (38). We suggest centrifugation at 2,500 x g for 10 minutes, based on the typical parameter values observed in the contributing EPHect centers, and in line with the UK Biobank. We recommend cooled (4°C) centrifugation as standard to avoid any effect of temperature on unknown biomarker stability. It is key for each center to apply centrifuge parameters of duration, speed, and temperature consistently across all samples processed; record these; and report them in all publications derived from the samples.

#### Long-term storage

The number and volume of the sample aliquots created should strike a balance between minimizing future freeze–thaw cycles and use of freezer space. Repeated freeze–thaw cycles are detrimental to the stability of biomolecules in some samples and should be avoided by creating multiple small-volume aliquots (100–500 μl) upfront before freezing 41, 42. If freezer space is limited, initial aliquots can be made in larger volumes (1.8–4.5 ml) and then aliquoted into smaller volumes the first time the aliquot is accessed.

Stability studies for a range of biomolecules have shown that samples should be stored as a minimum requirement in −80°C mechanical freezers for long-term storage (36). In the 1990s, a study showed that, depending on the location of the sample in the mechanical 80°C freezers, the actual temperature can fluctuate between −90°C and −43.5°C (43).

Liquid nitrogen freezers, which are colder and have less temperature variability than mechanical freezers (34), are recommended for standard long-term sample storage; however, they are more expensive and require access to a regular LN_2_ source. If several LN_2_ freezers are in the same room, oxygen sensors are required. Furthermore, −80°C and LN_2_ freezers need to be manually checked at least twice a week for temperature variations, and every freezer should be equipped with an alarm system to detect such variation. In addition, it is important to: [1] split samples from the same individual between freezers in case of a freezer malfunction, and [2] have an empty backup freezer available if possible. Freezers must be connected to power generators to ensure continued functioning during a power emergency, and to battery backups to protect them from power variations. An emergency plan must be designed that clearly specifies responsibilities and tasks to personnel if samples need to be moved to backup freezers.

### Urine

Urine has been widely used in metabolomic and proteomic studies for biomarker, hormone, and related metabolite detection 37, 44, 45 because of its easy, non-invasive collection in large quantities (46). A potential disadvantage is the unknown relevance of the molecules excreted in urine to the disease of interest. Furthermore, creatinine needs to be measured in samples to determine urine concentration, as this varies substantially within individuals over time (47). Six EPHect centers provided urine-sample SOPs.

#### Sample collection

The presence of host cells (e.g., harboring viruses) or bacterial cells in urine is a potential source of contamination that can influence the metabolic profile (37). Therefore, a “clean catch” protocol for sample collection is preferred, as it reduces the incidence of cellular and microbial contamination. The timing of sample collection for urine is complex, because each urine sample reflects what was metabolized and excreted since the previous void. The most comprehensive protocol collects all urine voided over a 24-hour period, which reflects excretion over the course of 1 day. However, this protocol may be unacceptable to some participants, or impossible for logistical or budgetary reasons. As an alternative, an overnight collection (collecting all urine from bedtime to the first morning void) may be preferred. First morning void samples can be collected, which represent the overnight period unless the participant voided during the night (23) and are better than a “spot urine” sample collected at a random time during the day 26, 46. If spot urines are collected, information should be collected on the timing of the last pre-collection void. The WERF EPHect standard recommendation is to collect clean catch, first morning void samples, with time of last food/drink consumption, any night voiding, and time of sample collection, recorded using the EPHect Biospecimen Form (Supplemental Appendix 7).

#### Sample stability, processing, and storage

The EPHect standard recommendation is to maintain urine samples at 4°C (or on ice) until processing and storage, to reduce the effects of possible enzymatic/cellular activities (37), and to store within 2 hours of collection. If first morning void urine samples are collected, the participant should keep the collected sample in the refrigerator and either bring the sample on ice to the clinic, or ship it on ice overnight, in which case the sample should be processed and stored within a maximum of 48 hours (28). Long-term storage of urine samples should ideally be in LN_2_ freezers or in −80°C freezers (see blood storage section).

### Saliva

Saliva samples are often used for DNA studies when blood sampling is not desirable or feasible (48), although an obstacle is that the sample can be contaminated with bacterial DNA (49). Saliva can also be used to measure other biomolecules such as hormones, with the limitation that only free (unbound) hormones are present and thus the concentrations are relatively low (50). Four EPHect centers provided saliva SOPs.

#### Sample collection

Several methods are available for collecting saliva for DNA, including “swish and spit,” saliva collection kits for DNA (e.g., Oragene^®^, DNAgard^®^, Norgen^®^), or swabs. The swish and spit method or Oragene^®^ kits are recommended as standard in EPHect, providing the best DNA quality and yield 51, 52, 53. For general biomarker studies, the “passive drool” method for sample collection is preferred over other methods that stimulate saliva production (e.g., chewing on cotton), as the latter can alter hormone levels (50). In addition, actively spitting has been shown to tighten muscles and may affect flow rate and concentration of proteins in saliva 54, 55. The amount of saliva collected is important for DNA yield (56). We recommend collection of 2 ml of saliva as the standard, with 1 ml as the minimum amount. To encourage participants to provide a sufficient sample, they can be shown pictures that can visually stimulate saliva production (e.g., pictures of lemons). Timing of saliva collection may be important, particularly if measuring stress biomarkers (46) and collecting time/date information are critical. In addition, it is important to record when the participant last brushed their teeth; chewed gum; smoked; or consumed alcohol, spicy food, or fishy food within the last 24 hours, as these can affect sample quality (see EPHect Biospecimen Form, Supplemental Appendix 7).

#### Sample stability, processing, and storage

Some salivary hormones are relatively stable in samples kept at room temperature for up to 1 week, although contamination with mold can be problematic. Thus, EPHect recommends keeping the sample chilled (4°C) 57, 58. For extraction of DNA using commercial saliva collection kits, the product instructions should be followed. Long-term storage should be in −80°C freezers as a minimum requirement, or in LN_2_ freezers per standard (see blood storage section).

### Peritoneal Fluid

Peritoneal fluid, present in the abdominal/pelvic cavity, reflects its specific microenvironment and has been used by a number of studies to investigate the roles of various constituent molecules in relation to endometriosis 59, 60, 61. Peritoneal fluid volume increases during the follicular phase of the menstrual cycle and decreases thereafter (60). Eight EPHect centers contributed their SOPs for peritoneal fluid collection.

#### Sample collection

During laparoscopy, after entry into the pelvic cavity, the peritoneal fluid is aspirated using a syringe or suction device (20). If no or very limited peritoneal fluid is found, a lavage method can be used to wash the peritoneal surfaces with 10 ml of sterile, normal saline solution using a laparoscopic needle, and manual aspiration can be performed using a syringe. This peritoneal lavage fluid (PLF) can be processed as peritoneal fluid, but the supernatant from PLF should be regarded with caution, as molecular profiles may vary depending on the collection method used. This method should be recorded (EPHect Biospecimen Form, Supplemental Appendix 7), along with menstrual data, as cycle phase may affect the concentration of molecules measured (61). Pilot studies are needed that compare the peritoneal microenvironments when sampling is performed using these different aspiration and lavage methods.

#### Sample stability, processing, and storage

The sample should be kept cool (on wet ice/at 4°C), and the processing time should be kept to a minimum to minimize degradation of molecules. The collected peritoneal fluid should be centrifuged in the laboratory, and the supernatant and the pellet (the cell fraction) should be stored separately, per standard, in LN_2_ freezers set at −80°C or lower (see blood storage section).

### Endometrial Fluid and Menstrual Effluent

Endometrial fluid is found in the endometrial cavity in the uterus 62, 63 and reflects its specific microenvironment. Menstrual effluent has been used for investigating molecules in menstruation/endometrium-related processes, such as angiogenesis and endometrial repair (64). Six EPHect centers provided endometrial fluid SOPs; 3 provided menstrual effluent SOPs.

#### Sample collection

Collection of an endometrial fluid sample is possible and advisable without administration of any pre-medication or anesthetics, since it is unknown if, and how quickly, medications can alter the expression of molecules in this microenvironment. If pre-medication or anesthetics are used, a record should be made of time of sample collection relative to administration. Endometrial fluid should not be collected during the menstrual cycle phase. Endometrial fluid is typically collected through an embryo-transfer catheter connected to a syringe 20, 63. If fluid volume is insufficient for the research purpose, a uterine lavage can be performed through slow infusion and withdrawal of 4 ml of normal, sterile saline solution into the uterine cavity (65). This uterine lavage fluid (ULF) can be processed as endometrial fluid, but the supernatant from ULF should be regarded with caution.

When comparing protein profiles of endometrial fluid collected using these two sampling techniques (66), both proved to be satisfactory sampling methods that enabled subsequent analysis of uterine fluid components. However, they provided substantially different protein profiles. The method of collection therefore needs to be recorded (EPHect Biospecimen Form, Supplemental Appendix 7).

Menstrual effluent is collected during the menstrual phase with a diaphragm or mixing cannula (64). For informative analysis of both endometrial fluid and menstrual effluent, menstrual cycle data should be recorded using the EPHect Biospecimen Form (Supplemental Appendix 7).

#### Sample stability, processing, and storage

The recommendation from EPHect is that endometrial fluid samples be kept cool (on wet ice/at 4°C) during processing and transferred to a screw top vial before centrifugation, with the supernatant and pellet stored separately. If the volume of the sample is not large enough for centrifugation, i.e., was collected with an embryo-transfer cannula, the cannula can be snap-frozen immediately in LN_2_/dry ice. For long-term storage, samples should, per standard, be stored in LN_2_ freezers set at −80°C or lower (see blood storage section).

### Biospecimen Form

The EPHect Biospecimen Form includes data items that the WERF EPHect Working Group agreed were essential to record from the participant when collecting biological samples. As mentioned above, data relevant to fluid biospecimen collections included an assessment of menstrual phase on the day of sample collection because various molecules are likely to be expressed at different levels in different phases of the menstrual cycle 67, 68, 69, 70, 71. Regularity of a participant's typical menstrual cycle should be recorded, along with LMP and ideally—through follow-up—the first day of the next menstrual cycle 72, 73, to allow accurate calculation of the cycle day on the day of sample collection. The EPHect Biospecimen Form also includes questions concerning parameters surrounding urine collection. EPHect recommends as standard the universal collection of samples *before* administration of any pre-medication or anesthetics where possible, as these could interfere with downstream molecular analyses, and any medication a participant has taken recently should be recorded.

The form also includes places to record weight, height, and waist and hip circumference, as measured by a research nurse. The EPHect Working Group agreed that these data are crucial to record because of the consistent phenotypic and genetic associations of obesity-related traits with endometriosis (74). It is critical that the anthropometric measurements are made using a method standardized both within and between studies, particularly for traits prone to measurement variability such as waist and hip circumference. We recommend the use of the National Health and Nutrition Examination Survey (NHANES) III guidelines (adapted from World Health Organization guidelines) for measuring waist and hip circumference 75, 76, described in Supplemental Appendix 8, available online.

## Discussion

We have provided WERF EPHect consensus SOPs for the collection of blood, urine, saliva, endometrial fluid, peritoneal fluid, and menstrual effluent in endometriosis research, together with a Biospecimen Form to collect additional data required for informative analysis of the samples. This consensus was developed and agreed on by 34 clinical/academic institutions and 3 industry collaborators from 16 countries across 5 continents. Adoption of the SOPs, and of the surgical and clinical data collection instruments described in our previous articles 20, 21, represents a ground-breaking opportunity for endometriosis research centers to decrease variability in—and increase validity of—their results, and to allow new comparisons and collaborations among centers 1, 19.

The SOPs presented focus on downstream analysis of biomolecules such as DNA, RNA, proteins, and metabolites, with specific relevance to endometriosis research. They may not be suited to the quantification of environmental chemicals in the sample, which may require different collection equipment as well as different SOPs. Although we focus on relevance to endometriosis research, these SOPs are clearly also relevant to research into other female conditions likely to use fluid samples that are subject to cyclical reproductive influences.

Although the consensus SOPs were based on the best evidence available, there were steps for which this evidence was limited. Specific evidence is lacking on the most effective duration, speed, and temperature conditions for centrifugation of blood samples. More pilot studies are needed on how the lavage methods used in both endometrial and peritoneal fluid collection affect downstream results for specific molecules.

All questionnaires and SOPs produced by the WERF EPHect Working Group are freely available for use by investigators, subject to signed, written, informed consent obtained from each patient, and local ethical approval for the study according to ethical principles for clinical research summarized in the Declaration of Helsinki. To enable the multi-center collaborations, envisaged by the WERF EPHect initiative, it is essential that centers adopting the WERF EPHect instruments and SOPs ensure that patients provide informed consent that allows their data and biological samples to be used in future multi-center (inter)national collaborations, and that appropriate ethics committee and institute review board approval is obtained.

The evidence base for all EPHect data collection instruments and SOPs will be reviewed continuously based on feedback provided by investigators, and through systematic surveys and follow-up reviews after 1 year, and every 3 years thereafter. Thus, investigators are strongly encouraged to provide such feedback. Updates of instruments will remain freely accessible to the research community through the WERF EPHect website (endometriosisfoundation.org/ephect). We ask that publication of results that are generated using WERF EPHect data and SOPs appropriately reference the sources, including version numbers, of the instruments used. In the next phase of the EPHect initiative, WERF aims to amalgamate a voluntary registry of centers using EPHect data collection tools and biological sample SOPs that would offer any investigator a transparent platform for establishing new collaborations.

Progress in other disease fields has shown that substantial advances can be made, particularly in terms of disease classification and biomarker identification, if biological sample collection from multiple sites can be combined; to this end, sample size and validity can be maximized through the use of standardized ongoing, long-term participant enrollment, sample collection, and storage. In addition, WERF EPHect envisages that adoption of the recommended standardized procedures will allow such significant advances to be made in the field of endometriosis, opening up new opportunities for international collaborations between academic as well as industry endometriosis research centers, and shedding new light on the etiology and methods for non-invasive diagnosis of this heterogeneous, enigmatic disease.
