# Supplementary material for: World Endometriosis Research Foundation Endometriosis Phenome and Biobanking Harmonization Project: III. Fluid biospecimen collection, processing, and storage in endometriosis research
Source: Fertil Steril. 2014 Nov;102(5):1233–43. doi: 10.1016/j.fertnstert.2014.07.1208 (PMC4230639; doi:10.1016/j.fertnstert.2014.07.1208)
Supplement: Supplemental Table 1 [file mmc1.docx]

**Supplemental Table 1:**

**VISUAL SUMMARY OF STANDARD OPERATING PROCEDURES FOR THE COLLECTION, PROCESSING, AND STORAGE OF BLOOD SPECIMEN**

|  | **Standard Recommended** | **Required Minimum** |
| --- | --- | --- |
| **Specimen**  **collection** | - Timing: before pre-med. - Fasting ≥ 10 hours. - Label collection tubes with 2D barcode and human readable labels. - 8-10 gentle inversions of the tube then place upright in a rack. | - Record whether sample is collected before or after pre-med/after anaesthesia. - Record the time of fasting. - Label collection tubes with human readable labels. - 8-10 gentle inversions of the tube then place upright in a rack. |
| **Specimen**  **processing** | - Temp: Within 1 hr→ keep at room temperature.   1hr- max. 4hr→ keep on wet ice/in refrigerator.   - Prioritize *EDTA* then *SST* collection then others. - Centrifuge: 10 min.* 2500g * 4°C | - Temp: Within 2 hr→ keep at room temperature.   2hr- max.4hr→ keep on wet ice/in refrigerator   - No priority given. - Centrifuge: 10 min.* 2500g * RT. |
| **Storage** | **Within max. 1 hour at LN_2_ freezer**  *Plasma/serum* → Gently aspirate the supernatant.   - Use screw-top gasket vials. - Aliquot on wet ice in upright position. - Aliquot volume: 100-500µl.   *WBCs* → Gently aspirate the buffy coat.   - Follow the same steps as for the supernatant.   *RBCs* → Gently mix RBCs then aspirate   - Follow aliquoting steps as for the supernatant. | **Within max. 4 hours at -80°C freezer**  *Plasma/serum* → Gently aspirate the supernatant.   - Use screw-top gasket vials. - Aliquot at room temp in upright position - Aliquot volume: 100-500µl.   *WBCs* → Gently aspirate the buffy coat.   - Follow the same steps as for the supernatant.   *RBCs* → Gently mix RBCs then aspirate   - Follow aliquoting steps as for the supernatant. |
| **Labelling** | Centre:  Participant ID:  Aliquot ID:  Sampling date:  Sample type: 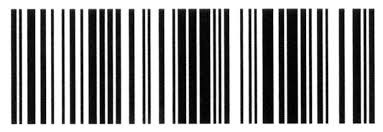 | Centre:  Participant ID:  Aliquot ID:  Sampling date:  Sample type: |
| **Freezer**  **check** | - Store sample aliquots in separate freezers. - Alarm system setup on all freezers. - Biweekly human check. | - Biweekly human check. |
| **Sample**  **Long-term log** | - Record any freeze-thaw cycles. - Track change in sample location or consumption. - Track new samples from original aliquots. | |
| **Check list data recording** | - Time of last eating/drinking except plain water. - Date/time of sample collection. - Start time of sample processing. - Number/volume/type of aliquots. - Date/time aliquot storage. - Record variations or deviations of the sample character. - Log of any freeze-thaw of aliquots. - Biweekly log of freezer check. | |
