# Supplementary material for: World Endometriosis Research Foundation Endometriosis Phenome and Biobanking Harmonization Project: III. Fluid biospecimen collection, processing, and storage in endometriosis research
Source: Fertil Steril. 2014 Nov;102(5):1233–43. doi: 10.1016/j.fertnstert.2014.07.1208 (PMC4230639; doi:10.1016/j.fertnstert.2014.07.1208)
Supplement: Supplemental Table 2 [file mmc2.docx]

**Supplemental Table 2:**

**VISUAL SUMMARY OF STANDARD OPERATING PROCEDURES FOR THE COLLECTION, PROCESSING, AND STORAGE OF URINE SPECIMEN**

|  | **Standard Collection** | **Required minimum** |
| --- | --- | --- |
| **Specimen**  **collection** | - A clean catch mid-stream first morning void urine when they get out of bed in a sterile container. - Put in a refrigerator and deliver in an ice pack to the clinic (4°C). - Record the time of fasting - Record time of first morning void and whether the participant urinated during the night. - Label collection tubes with 2D barcode and human readable labels. | - A clean catch spot urine sample from the patient in the clinic or at the patient’s home in a sterile container. - Put in refigerator and deliver in an ice pack, if collected in clinic directly put on wet ice (4°C). - Record the time of fasting - Record time of spot urine collection. - Label collection tubes with human readable labels. |
| **Specimen**  **processing** | - Within 2 hours. - Discard the sample if there is blood in it. - Mix the sample. - Perform dipstick urinalysis for specific gravity. - Centrifuge at 1000-3000g at 4°C for 5 minutes. | - Within a maximum of 48 hours. - Discard the sample if there is blood in it. - Mix the sample. - Perform dipstick urinalysis for specific gravity. - Centrifuge at 1000-3000g at 4°C for 5 minutes. |
| **Storage** | **Within max. 2 hour at LN_2_ freezer**   - Unprocessed sample → store in LN_2_ freezer. - Processed sample → place the sample on wet ice and aspirate the supernatant into required number of aliquots → store in LN_2_ freezer. | **Within max. 48 hours at -80°C freezer**   - Unprocessed sample → store at -80°C freezer. - Processed sample → place the sample on wet ice and aspirate the supernatant into required number of aliquots → store at -80°C freezer. |
| **Labelling** | Centre:  Participant ID:  Aliquot ID:  Sampling date:  Sample type: 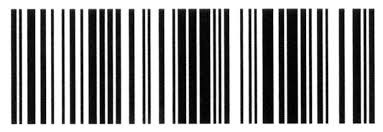 | Centre:  Participant ID:  Aliquot ID:  Sampling date:  Sample type: |
| **Freezer**  **check** | - Store sample aliquots in separate freezers. - Alarm system setup on all freezers. - Biweekly human check. | - Biweekly human check. |
| **Sample**  **Long-term log** | - Record any freeze-thaw cycles. - Track change in sample location or consumption. - Track new samples from original aliquots. | |
| **Check list data recording** | - Time of last eating/drinking except plain water. - Date/time of sample collection. - Start time of sample processing. - Record the results of dipstick for specific gravity. - Number/volume/type of aliquots. - Date/time aliquot storage. - Record variations or deviations of the sample character. - Log of any freeze-thaw of aliquots. - Biweekly log of freezer check. | |
