# Supplementary material for: World Endometriosis Research Foundation Endometriosis Phenome and Biobanking Harmonization Project: III. Fluid biospecimen collection, processing, and storage in endometriosis research
Source: Fertil Steril. 2014 Nov;102(5):1233–43. doi: 10.1016/j.fertnstert.2014.07.1208 (PMC4230639; doi:10.1016/j.fertnstert.2014.07.1208)
Supplement: Supplemental Table 3 [file mmc3.docx]

**Supplemental Table 3:**

**VISUAL SUMMARY OF STANDARD OPERATING PROCEDURES FOR THE COLLECTION, PROCESSING, AND STORAGE OF SALIVA SPECIMEN**

|  | **Standard Collection** | **Required minimum** |
| --- | --- | --- |
| **Specimen**  **collection** | - Fasting ≥6 hours. - Rinse mouth. - Drooling NOT spitting. - No salivary stimulants. - 2ml Saliva (avoid bubbles). - Label collection tubes with 2D barcode and human readable labels. | - Brushing tooth/eating meal >1 hours. - Alcohol drink >12 hours. - Citric/sugary food >20 minutes. - Rinse mouth. - Spitting/drooling. - No salivary stimulants. - 1ml Saliva (avoid bubbles). - Label collection tubes with human readable labels. |
| **Specimen**  **processing** | - Temp: Within 1 hr→ keep at RT.   >1hr→ keep on wet ice/refrigerator.   - Centrifuge: 2 min.* 1000g * 4°C | - Temp: Within 1 hr→ keep at room temperature.   >1hr→ keep on wet ice/refrigerator.   - Centrifuge: 2 min.* 1000g * 4°C |
| **Storage** | **Within max. 4 hour in LN_2_ freezer**  Unprocessed sample → store in LN_2_ freezer.  Gently aspirate the supernatant   - Use screw-top gasket. - Aliquoting on wet ice in upright position.   *Saliva for RNA extraction:*   - Use RNA-stabilised aliquots and proceed as described in the product manual. | **Stored at -80°C freezer.**  Unprocessed sample → store at -80°C freezer.  Gently aspirate the supernatant.   - Use screw-top gasket. - Aliquot at room temperature in upright position.   *Saliva for RNA extraction:*   - Use RNA--stabilised aliquots and proceed as described in the product manual. - Record time from sample collection to storage. |
| **Labelling** | Centre:  Participant ID:  Aliquot ID:  Sampling date:  Sample type: 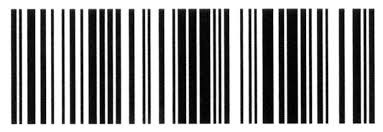 | Centre:  Participant ID:  Aliquot ID:  Sampling date:  Sample type: |
| **Freezer**  **check** | - Split samples on two separate freezers. - Alarm system setup on all freezers. - Biweekly human check. | - Biweekly human check. |
| **Sample**  **Long-term log** | - Record any freeze-thaw cycles. - Track change in sample location or consumption. - Track new samples from original aliquots. | |
| **Check list data recording** | - Time of last eating/drinking except plain water. - Record last tooth brushing/citrus or sugary food/alcohol intake. - Date/time of sample collection. - Start time of sample processing. - Number/volume/type of aliquots. - Date/time aliquot storage. - Record variations or deviations of the sample character. - Log of any freeze-thaw of aliquots. - Biweekly log of freezer check. | |
