# Supplementary material for: World Endometriosis Research Foundation Endometriosis Phenome and Biobanking Harmonization Project: III. Fluid biospecimen collection, processing, and storage in endometriosis research
Source: Fertil Steril. 2014 Nov;102(5):1233–43. doi: 10.1016/j.fertnstert.2014.07.1208 (PMC4230639; doi:10.1016/j.fertnstert.2014.07.1208)
Supplement: Supplemental Table 4 [file mmc4.docx]

**Supplemental Table 4:**

**VISUAL SUMMARY OF STANDARD OPERATING PROCEDURES FOR THE COLLECTION, PROCESSING, AND STORAGE OF ENDOMETRIAL FLUID SPECIMEN**

|  | **Standard Collection** | **Required minimum** |
| --- | --- | --- |
| **Specimen**  **collection** | - Timing: before pre-med. - Use embryo catheter/Pipelle. - Label collection tubes with 2D barcode and human readable labels. | - Record whether sample is collected before or after pre-med/anaesthesia. - Use embryo catheter/Pipelle. - Label collection tubes with human readable labels. |
| **Specimen**  **processing** | - Centrifuge at 4°C for your Lab adapted SOP. | - Centrifuge at room temperature for your Lab adapted SOP. |
| **Storage** | **Store at LN_2_ freezer**  Unprocessed sample → store in LN_2_ freezer.  The supernatant → gently aspirate:   - Use screw-top gasket - Aliquot on wet ice and in upright position   The pellet→ use screw-top Gasket | **Store at -80°C freezer**  Unprocessed sample → store at -80°C freezer.  The supernatant → gently aspirate:   - Use screw-top gasket - Aliquot at room temperature and in upright position   The pellet→ use screw-top Gasket |
| **Labelling** | Centre:  Participant ID:  Aliquot ID:  Sampling date:  Sample type: 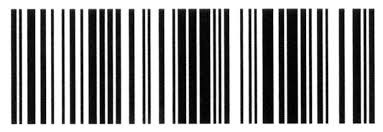 | Centre:  Participant ID:  Aliquot ID:  Sampling date:  Sample type: |
| **Freezer**  **check** | - Store aliquots in separate freezers. - Alarm system setup on all freezers. - Biweekly human check. | - Biweekly human check. |
| **Sample**  **Long-term log** | - Record any freeze-thaw cycles. - Track change in sample location or consumption. - Track new samples from original aliquots. | |
| **Check list data recording** | - Time of last eating/drinking except plain water. - Date/time of sample collection. - Start time of sample processing. - Number/volume/type of aliquots. - Date/time aliquot storage. - Record variations or deviations of the sample character. - Log of any freeze-thaw of aliquots. - Biweekly log of freezer check. | |
