# Supplementary material for: World Endometriosis Research Foundation Endometriosis Phenome and Biobanking Harmonization Project: III. Fluid biospecimen collection, processing, and storage in endometriosis research
Source: Fertil Steril. 2014 Nov;102(5):1233–43. doi: 10.1016/j.fertnstert.2014.07.1208 (PMC4230639; doi:10.1016/j.fertnstert.2014.07.1208)
Supplement: Supplemental Table 5 [file mmc5.docx]

**Supplemental Table 5:**

**VISUAL SUMMARY OF STANDARD OPERATING PROCEDURES FOR THE COLLECTION, PROCESSING, AND STORAGE OF PERITONEAL FLUID SPECIMEN**

|  | **Standard Collection** | **Required minimum** |
| --- | --- | --- |
| **Specimen**  **collection** | - Timing: after anaesthesia. - Sample collection is performed by 20ml suction devices /laparoscopic needle. - If no PF→ peritoneal lavage with 20ml normal saline (use the pellet, discard the supernatant). - Labelling → Standard: Human readable and 2D barcode labels   → Minimum: Human readable label | |
| **Specimen**  **processing** | - Transfer to the lab within 30 minutes. - Centrifuge at 4°C. | - Transfer to the lab as soon as possible. - Centrifuge at room temperature. |
| **Storage** | **Store at LN_2_ freezer**  The supernatant → gently aspirate:   - Use screw-top gasket - Aliquot on wet ice and in upright position.   *Discard the supernatant if peritoneal lavage!*  The pellet→ use screw-top gasket | **Store at -80°C freezer**  The supernatant → gently aspirate:   - Use screw-top gasket - Aliquot at room temperature and in upright position   *Discard the supernatant if peritoneal lavage!*  The pellet→ use screw-top gasket |
| **Labelling** | Centre:  Participant ID:  Aliquot ID:  Sampling date:  Sample type: 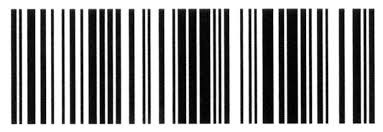 | Centre:  Participant ID:  Aliquot ID:  Sampling date:  Sample type: |
| **Freezer**  **check** | - Store aliquots in separate freezers. - Alarm system setup on all freezers. - Biweekly human check. | - Biweekly human check. |
| **Sample**  **Long-term log** | - Record any freeze-thaw cycles. - Track change in sample location or consumption. - Track new samples from original aliquots. | |
| **Check list data recording** | - Time of last eating/drinking except plain water. - Date/time of sample collection. - Start time of sample processing. - Number/volume/type of aliquots. - Date/time aliquot storage. - Record variations or deviations of the sample character. - Log of any freeze-thaw of aliquots. - Biweekly log of freezer check. | |
