# Supplementary material for: World Endometriosis Research Foundation Endometriosis Phenome and Biobanking Harmonization Project: III. Fluid biospecimen collection, processing, and storage in endometriosis research
Source: Fertil Steril. 2014 Nov;102(5):1233–43. doi: 10.1016/j.fertnstert.2014.07.1208 (PMC4230639; doi:10.1016/j.fertnstert.2014.07.1208)
Supplement: Supplemental Table 6 [file mmc6.docx]

**Supplemental Table 6:**

**VISUAL SUMMARY OF STANDARD OPERATING PROCEDURES FOR THE COLLECTION, PROCESSING, AND STORAGE OF MENSTRUAL EFFLUENT (BLOOD) SPECIMEN**

|  | **Standard Collection** | **Required minimum** |
| --- | --- | --- |
| **Specimen**  **collection** | - Collect menstrual effluent sample with a diaphragm or mixing cannula. - Labelling → Standard: Human readable and 2D barcode labels   → Minimum: Human readable label | |
| **Specimen**  **processing** | - Within 1 hour on wet ice. - For plasma→ use EDTA tubes on wet ice. - For cells → use heparin tubes on wet ice. - Centrifuge: 10 min.* 2500g * 4°C | - Within 1 hour at room temperature - For plasma→ use EDTA tubes on wet ice. - For cells → use heparin tubes on wet ice. - Centrifuge: 10 min.* 2500g * 4°C |
| **Storage** | **Within max. 1 hour**  Unprocessed sample → store in LN_2_ freezer.  *For Plasma/serum* → gently aspirate the supernatant   - Use screw-top gasket - Aliquot on wet ice and in upright position   *For cells*→ collect at the interphase for culture or flow cytometry. | **Within max. 1 hour**  Unprocessed sample → store at -80°C freezer.  *For Plasma/serum* → gently aspirate the supernatant   - Use screw-top gasket - Aliquot on wet ice and in upright position   *For cells*→ collect at the interphase for culture or flow cytometry. |
| **Labelling** | Centre:  Participant ID:  Aliquot ID:  Sampling date:  Sample type: 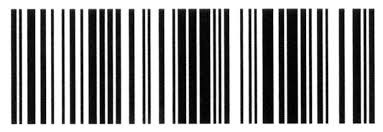 | Centre:  Participant ID:  Aliquot ID:  Sampling date:  Sample type: |
| **Freezer**  **check** | - Store aliquots in separate freezers. - Alarm system setup on all freezers. - Biweekly human check. | - Biweekly human check. |
| **Sample**  **Long-term log** | - Record any freeze-thaw cycles. - Track change in sample location or consumption. - Track new samples from original aliquots. | |
| **Check list data recording** | - Time of last eating/drinking except plain water. - Date/time of sample collection. - Start time of sample processing. - Number/volume/type of aliquots. - Date/time aliquot storage. - Record variations or deviations of the sample character. - Log of any freeze-thaw of aliquots. - Biweekly log of freezer check. | |
