# Supplementary material for: World Endometriosis Research Foundation Endometriosis Phenome and Biobanking Harmonization Project: III. Fluid biospecimen collection, processing, and storage in endometriosis research
Source: Fertil Steril. 2014 Nov;102(5):1233–43. doi: 10.1016/j.fertnstert.2014.07.1208 (PMC4230639; doi:10.1016/j.fertnstert.2014.07.1208)
Supplement: Supplemental Appendix 1 [file mmc7.docx]

**Supplemental Appendix I:**

**Detailed standard operating procedure for the collection, processing and storage of blood specimens**

**NOTES**

- This SOP applies to plasma collection using EDTA or lithium-heparin coagulation tubes, and serum with (SST) or without separator, collected via venepuncture.
- This SOP does not cover blood withdrawal techniques/procedures (qualified personnel should follow standard withdrawal procedures).
- This SOP does not cover safety procedures for the collection and processing of these samples and personnel must follow institutional biosafety guidelines**.**
- For a summary version of this protocol with side-by-side standard vs. minimal protocol step comparisons, please see Supplemental Table 1.
- As this protocol applies to different processing and storage methods (e.g. the use of RNA stabilising fluid), keep a copy of the exact step-by-step protocol used in your lab.

***Processing and storage materials***

1. Biospecimen form (Supplemental Appendix VII);
2. Log sheet to record sample-related data;
3. Blood collection tubes with EDTA and heparin for plasma, SST or no SST for serum
4. Crushed ice if a delay is anticipated
5. Appropriate racks to hold tubes in upright position
6. Centrifuge
7. Volume adjustable pipette
8. Transfer pipettes
9. Labels suitable for long-term freezer storage, and IDs printed using 2D barcoding
10. Aliquot vials with screw top gasket closure
11. Freezers: -80C or liquid nitrogen (LN_2_)

**1. Blood collection**

1.1. Blood collection should be performed by a licensed phlebotomist, nurse, anaesthesiologist, or medical doctor.

1.2. Collection should be performed in an adequate setting, e.g. in the phlebotomy room, or on the ward. Blood collection in the operating theatre should be avoided, if possible.

1.3. Time of sample collection in the clinic:

1.3.1. ***Standard collection:*** Collect blood samples before induction of anaesthesia (before pre-med is given).

1.3.2. ***Required minimum:*** Record whether blood is collected (1) prior to pre-medication; (2) after pre-medication but before anaesthesia; or (3) after anaesthesia.

1.4. Fasting status at sample collection:

1.4.1. ***Standard collection:*** Collect only samples after fasting for at least 10 hours. Record on the log sheet the time since the study participant ate or drank anything except plain water (Fasted since: __:__pm/am).

1.4.2. ***Required minimum:*** Record on the log sheet the time since the study participant ate or drank anything except plain water (Fasted since: __:__pm/am).

1.5. Preparation of sample collection tubes:

1.5.1. ***Standard collection:*** Label each blood collection tube with a 2D barcode in addition to a human readable unique identifier, participant ID, date of collection and type of sample. Record on the log sheet the date and time of sample collection (Date: __/__/__ and __:__am/pm).

1.5.2. ***Required minimum:*** Label each blood collection tube with a unique identifier, participant ID, date of collection, and type of sample. Record on the log sheet the date and time of sample collection (Date: __/__/__ and __:__am/pm).

1.6. Order of sample type collection:

1.6.1. ***Standard collection:*** Prioritise: 1) EDTA plasma; 2) SST serum; 3) other tube types, in a pre-determined order of priority. Keep a record of the order in your adapted SOP.

1.6.2. ***Required minimum:*** Collect collection tubes always in the same order of priority and keep a record of the order in your adapted SOP.

1.7. It is important that tubes with anti-coagulants (e.g., EDTA, heparin), which need to be inverted after blood draw, are gently inverted 8-10 times (no vigorous shaking). Place in the collection rack in an upright position.

1.8. Temperature and waiting conditions of samples until processed in the lab:

1.8.1. ***Standard collection:*** Place samples on wet ice/in refrigerator immediately if there will be more than 1 hour before processing (maximum time to processing: 4 hours). If the time to processing is less than 1 hour, samples can be kept at room temperature.

1.8.2. ***Required minimum:*** Place samples on wet ice/in refrigerator if there will be more than 2 hour before processing (maximum time to processing: 4 hours). If the time to processing is less than 2 hours, samples can be kept at room temperature.

**2. Sample processing in the laboratory**

2.1. Allow any SST tubes (red top) to clot for 30-60 minutes in an upright position at room temperature in the first 30 minutes from collection of the sample. SST tubes should be placed on wet ice after 1 hour if there will be more than 2 hours until processing.

2.2. Time until samples processed in the lab:

2.2.1. ***Standard collection:*** Record on the log sheet the time sample processing started in the laboratory. Blood samples should be centrifuged within 1 hour of blood collection.

2.2.2. ***Required minimum:*** Record on the log sheet the time sample processing started in the laboratory. Blood samples should be centrifuged within 4 hours of blood collection.

2.3. Centrifugation of samples:

2.3.1. ***Standard collection:*** Centrifuge samples for 10 minutes at 2500g at 4°C. Keep a record of the standard time and g in your centre’s SOP.

2.3.2. ***Required minimum:*** Centrifuge samples for 10 minutes at 2500g at room temperature. Keep a record of the standard time and g in your centre’s SOP.

2.4. After centrifugation of samples:

2.4.1. ***Standard collection:*** Place the spun tubes on a rack in upright position and on wet ice during aliquotting.

2.4.2. ***Required minimum:*** Place the spun tubes on a rack in upright position at room temperature during aliquotting.

2.5. Aliquotting of samples:

2.5.1. ***Standard collection:*** Have a set number of aliquot tubes and sizes for each sample type that will be collected. Pre-label aliquot vials (see section 3). Put empty aliquot vials into a rack that sits on wet ice before aliquotting. Aliquot into small volumes between 100-500uL to minimize later freeze-thaw cycles.

2.5.2. ***Required minimum:*** Have a set number of aliquot tubes and sizes for each sample type that will be collected. Pre-label aliquot vials (see section 3).

2.6. Aspirate plasma/serum using an appropriate transfer pipette (ideally a volume-adjustable pipette) being careful not to disturb the cell layer below, holding the tube at a 45° angle.

2.7. Transfer plasma/serum to appropriately sized aliquot vial with screw top gasket closure (do not use tubes with a push top as they are not airtight) and fill as close to full as possible to minimise exposure to air.

2.8. Repeat steps 2.6 and 2.7 until all the plasma and serum has been transferred. Record volume of sample in each aliquot of plasma or serum.

2.9. If cells are accidentally mixed with the plasma/serum, the aliquot vial can be re-centrifuged as before and the plasma/serum can be transferred to a new aliquot vial.

2.10. White Blood Cell (WBC) aliquotting: Using a transfer pipette take the buffy coat layer from the collection tube. Aspirate slowly and carefully using a circular motion to remove all the visible buffy coat and transfer to an appropriate sized aliquot vial with screw top gasket closure. Record the volume of sample in each aliquot of WBC.

2.11. Red Blood Cell (RBC) aliquotting: Using a transfer pipette, gently mix the remaining erythrocytes and aspirate in another appropriate sized aliquot vial with screw top gasket closure and fill as close to full as possible to minimise surface area. Record the volume of sample in each aliquot of RBC.

**3. Labelling aliquots and storage**

3.1. Use special labels and ink that do not disintegrate when stored in very low temperature freezers (i.e. do not use laser printers or most ink-printers as they disintegrate when frozen).

3.2. Preparation of sample aliquot tubes:

3.2.1. ***Standard collection:*** Label the aliquot tubes with the participant ID number followed by a unique aliquot ID number. For example: ENDO-123456-U654321-P-01: Center identifier (ENDO), participant ID (123456), unique aliquot vial ID (U654321), sample type (P for plasma), aliquot number (01). Also, include date of sample creation on the label to be able to distinguish samples from the same participant collected at different time points. Furthermore, include the above information in human readable format and in a 2D barcode on the label.

3.2.2. ***Required minimum:*** Label the aliquot tubes with the participant ID followed by a unique aliquot ID number. For example: ENDO-123456-U654321-P-01: Center identifier (ENDO), participant ID (123456), unique aliquot vial ID (U654321), sample type (P for plasma), aliquot number (01). Also, include date of sample creation on the label to be able to distinguish samples from the same participant collected at different time points.

3.3. Time until sample aliquots are put into freezers for storage:

3.3.1. ***Standard collection:*** Samples should be processed and stored into freezers within a maximum of 1 hour and time should be recorded on the log sheet. Also record the type, number and volume of aliquots prepared.

3.3.2. ***Required minimum:*** Samples should be processed and stored into freezers within a maximum of 4 hours, and time of storage should be recorded on the log sheet. Also record the type, number and volume of aliquots prepared.

3.4. Sample storage in freezers:

3.4.1. ***Standard collection:*** Store serum, plasma and WBC/RBC aliquots in liquid nitrogen (LN_2_) freezers, which have less temperature fluctuations than -80°C freezers.

3.4.2. ***Required minimum:*** Store serum, plasma and WBC/RBC aliquots in -80°C or lower freezers.

3.5. Record on the log sheet any variations or deviations from the SOP, problems, or issues (e.g., hemolysis, vial cracked during processing).

3.6. Record the location of each sample in the freezer including freezer number, rack, box, and position in the box along with all other sample attributes in a database. If possible avoid using a spreadsheet format, but preferably use a relational database.

**4. Freezer check**

4.1.1. ***Standard collection:*** Split aliquots from the same sample type and individual between freezers in case of a freezer breaking down. Check freezers bi-weekly and keep a written-log of checks. Have alarm systems setup on all freezers in addition to human bi-weekly checks.

4.1.2. ***Required minimum:*** Manually check freezers bi-weekly and keep a written-log of checks.

**5. Data recording check list**

5.1. Record protocol, specifying which steps are adhered to (standard or minimum).

5.2. Record the time since the study participant ate or drank anything except plain water (fasted since: __:__pm/am).

5.3. For each sample, record:

5.3.1. Date and time of blood collection (Date: __/__/__ and __:__am/pm).

5.3.2. Start time of sample processing in the laboratory (__:__am/pm).

5.3.3. Type, number and volume of aliquots prepared.

5.3.4. Date and time aliquots stored into freezers (Date: __/__/__ and __:__am/pm).

5.3.5. Any variations or deviations from the SOP, problems, or issues.

5.4. In the long-term, record:

5.4.1. Any freeze-thaw that occurs with a sample for any reason.

5.4.2. Any change of location of a sample, including sending a sample out to an assay lab for processing.

5.4.3. Any new samples created from the original aliquots (i.e., a sub-aliquot) in the same manner as described above.

5.5. Keep a bi-weekly log of freezer checks.
