# Supplementary material for: World Endometriosis Research Foundation Endometriosis Phenome and Biobanking Harmonization Project: III. Fluid biospecimen collection, processing, and storage in endometriosis research
Source: Fertil Steril. 2014 Nov;102(5):1233–43. doi: 10.1016/j.fertnstert.2014.07.1208 (PMC4230639; doi:10.1016/j.fertnstert.2014.07.1208)
Supplement: Supplemental Appendix 2 [file mmc8.docx]

**Supplemental Appendix II:**

**Detailed standard operating procedure for the collection, processing and storage of urine specimens**

**NOTES**

- This SOP does not cover safety procedures for the collection and processing of these samples and personnel must follow institutional biosafety guidelines**.**
- For a summary version of this protocol with side-by-side standard vs. minimal protocol step comparisons, please see Supplemental Table 2.
- As this protocol applies to different processing and storage methods, keep a copy of the exact step-by-step protocol used in your lab.

***Processing and storage materials***

1. Biospecimen form (Supplemental Appendix VII);
2. Log sheet to record sample-related data;
3. Sterile urine collection container with a wide mouth and a leak-proof cap
4. Crushed ice if a delay is anticipated
5. Transfer pipette
6. Dipstick for urine analysis
7. Volume adjustable pipette
8. Centrifuge
9. Labels suitable for long-term freezer storage, and IDs printed using 2D barcoding
10. Aliquot vials with screw top gasket closure
11. Freezers: -80C or liquid nitrogen (LN_2_)

**1. Urine collection**

1.1. Sample collection method:

1.1.1. ***Standard collection:*** Obtain a clean catch, mid-stream, first morning void*, urine sample from the patient. Instruct them to collect the urine sample first thing in the morning when they get out of bed. Provide the patient with a sterile specimen container with a wide mouth and a leak-proof cap and written instructions on how to collect clean catch midstream urine sample.

1.1.2. ***Required minimum:*** Obtain a clean catch urine sample from the patient in the clinic or at the patient’s home by providing the patient with a sterile specimen container with a wide mouth and a leak-proof cap and written instructions on how to collect clean catch midstream urine sample.

*Patient instruction for first morning clean catch urine sample^[[1]](#footnote-1)^:

1. Collect the first urine that occurs as the first void after waking up.
2. Wash your hands with soap and warm water.
3. Sit on the toilet with your legs spread apart. Use two fingers to spread open your labia.
4. Use an antiseptic wipe to clean the inner folds of the labia. Wipe from the front to the back.
5. Use a second antiseptic wipe to clean over the opening where urine comes out (urethra), just above the opening of the vagina.
6. Keeping your labia spread open, urinate a small amount into the toilet bowl, then stop the flow of urine.
7. Hold the urine cup a few inches from the urethra and urinate until the cup is about half full.
8. You may finish urinating into the toilet bowl.
9. Screw the cap on to the collection cup securely and place in the refrigerator until taking the sample to the clinic.

1.2. If the sample is collected in the clinic, the research nurse should record the time of sample collection. If the sample is a first morning void brought into clinic with the patient, the patient should record the time and type of sample collection and report to the research nurse.

1.3. If the sample is collected in clinic, it should be put on wet ice immediately. If the patient collects the sample at home, it should be maintained in a refrigerator and delivered in an ice pack to the clinic (at 4°C).

1.4. Record on the log sheet the time since the study participant ate or drank anything except plain water (Fasted since: __:__pm/am). Record what time the urine sample was collected, whether this was a first morning void or spot urine, and whether the study participant has urinated during the night time if the collected sample is a first morning void.

1.5. Labelling of the collection cup:

1.5.1. ***Standard collection:*** Label the collection cup before giving to the patient for collection, with a 2D barcode in addition to human-readable the unique identifier of the patient and sample identifier.

1.5.2. ***Required minimum:*** Once the collection cup is collected from the patient, label the sample with the unique identifier of the patient and sample identifier.

**2. Sample processing in the laboratory, labelling aliquots and storage**

2.1. Time until samples processed in the lab:

2.1.1. ***Standard collection:*** Keep the sample refrigerated until processed and complete processing within 2 hours. Record on the log sheet the time sample processing started in the laboratory.

2.1.2. ***Required minimum:*** Keep the sample refrigerated until processed and complete processing within a maximum of 48 hours. Record on the log sheet the time sample processing started in the laboratory.

2.2. Discard the sample if it contains blood, and record.

2.3. Mix the sample by either swirling the cup or pipetting the urine up and down a couple of times.

2.4. Perform dipstick urine analysis and record result including specific gravity. If specific gravity is lower than 1.001 or greater than 1.032, retest for accuracy. Record on the log sheet, the results, including specific gravity and that retest is performed.

2.5. Aspirate required amount of unprocessed urine and label the aliquot vials with screw top gasket closures.

2.6. Preparation of sample aliquot tubes:

2.6.1. ***Standard collection:*** Label the aliquot vials with the participant ID number followed by a unique aliquot ID number. For example: ENDO-123456-U654321-U-01: Center identifier (ENDO), participant ID (123456), unique aliquot vial ID (U654321), sample type (U for urine) and aliquot number (01). Also, include date of sample creation on the label to be able to distinguish samples from the same participant collected at different time points. Further, include the above information in human readable format and in a 2D barcode on the label.

2.6.2. ***Required minimum:*** Label the aliquot vials with the participant ID followed by the sample aliquot number. Also include date of sample creation on the label. For example: ENDO-123456-U-01: Center identifier (ENDO), participant ID (123456), type of sample (U for urine), aliquot number (01). Also, include date of sample creation on the label to be able to distinguish samples from the same participant collected at different time points.

2.7. Sample storage in freezers:

2.7.1. ***Standard collection:*** Store the unprocessed urine aliquots in liquid nitrogen (LN_2_) freezers, which have less temperature fluctuations.

2.7.2. ***Required minimum:*** Store the unprocessed urine aliquots at -80°C or lower.

2.8. Fill a sterile tube with the remaining urine in the sample collection container and centrifuge at 1000-3000g at 4°C for 5 minutes.

2.9. Place the sample on wet ice and aspirate the supernatant into required number of aliquots. Label the aliquots as in 2.6. and store the processed urine aliquots as in 2.7.

2.10. Duration until sample aliquots are put into freezers for storage:

2.10.1. ***Standard collection:*** Samples should be processed and stored into freezers within a maximum of 2 hours and time should be recorded on the log sheet. Also record the type, number and volume of aliquots prepared.

2.10.2. ***Required minimum:*** Samples should be processed and stored into freezers within a maximum of 48 hours and time should be recorded on the log sheet. Also record the type, number and volume of aliquots prepared.

2.11. Record on the log sheet any variations or deviations from the SOP, problems, or issues.

2.12. Record the location of each sample in the freezer including freezer number, rack, box, and position in the box along with all other sample attributes in a database. If possible, avoid using a spreadsheet format, but preferably use a relational database.

**3. Freezer check**

3.1.2. ***Standard collection:*** Split aliquots from the same sample type and individual between freezers in case of a freezer breaking down. Check freezers bi-weekly and keep a written-log of checks. Have alarm systems setup on all freezers in addition to human bi-weekly checks.

3.1.1. ***Required minimum:*** Manually check freezers bi-weekly and keep a written-log of checks.

**4. Data recording check list**

4.1. Record protocol, specifying which steps are adhered to (standard or minimum).

4.2. Record the time since the study participant ate or drank anything except plain water (Fasted since: __:__pm/am).

4.3. For each sample, record:

4.3.1. Date and time of urine collection (Date: __/__/__ and __:__am/pm).

4.3.2. Type of urine collection (e.g. Clean catch spot urine or clean catch first morning void urine).

4.3.3. Start time of sample processing in the laboratory (__:__am/pm).

4.3.4. Results of dipstick urinalysis including specific gravity and that retest is performed.

4.3.5. Type, number and volume of aliquots prepared.

4.3.6. Date and time aliquots stored into freezers (Date: __/__/__ and __:__am/pm).

4.3.7. Any variations or deviations from the SOP, problems, or issues.

4.4. In the long-term, record:

4.4.1. Any freeze-thaw that occurs with a sample for any reason.

4.4.2. Any change of location of a sample, including sending a sample out to an assay lab for processing.

4.4.3. Any new samples created from the original aliquots (i.e., a sub-aliquot) in the same manner as described above.

4.5. Keep a bi-weekly log of freezer checks.

1. http://www.nlm.nih.gov/medlineplus/ency/article/007487.htm [↑](#footnote-ref-1)
