# Supplementary material for: World Endometriosis Research Foundation Endometriosis Phenome and Biobanking Harmonization Project: III. Fluid biospecimen collection, processing, and storage in endometriosis research
Source: Fertil Steril. 2014 Nov;102(5):1233–43. doi: 10.1016/j.fertnstert.2014.07.1208 (PMC4230639; doi:10.1016/j.fertnstert.2014.07.1208)
Supplement: Supplemental Appendix 3 [file mmc9.docx]

**Supplemental Appendix III:**

**Detailed standard operating procedure for the collection, processing and storage of saliva specimens**

**NOTES**

- This SOP does not cover safety procedures for the collection and processing of these samples and personnel must follow institutional biosafety guidelines**.**
- For a summary version of this protocol with side-by-side standard vs. minimal protocol step comparisons, please see Supplemental Table 3.
- As this protocol applies to different processing and storage methods, keep a copy of the exact step-by-step protocol used in your lab.

***Processing and storage materials***

1. Biospecimen form (Supplemental Appendix VII);
2. Log sheet to record sample-related data;
3. Sterile saliva collection container or is collecting sample for DNA the manufacturer’s provided collection container
4. Crushed ice if a delay is anticipated.
5. Transfer pipette
6. Volume adjustable pipette
7. Centrifuge
8. RNA stabilizer fluid *(optional if planning RNA studies)*
9. Labels suitable for long-term freezer storage, and IDs printed using 2D barcoding
10. Aliquot vials with screw top gasket closure
11. Freezers: -80C or liquid nitrogen (LN_2_)

**1. Saliva collection**

1.1. Record whether the patient has fasted and for how long.

1.2. Record recent (last 24 hours) exposures to toothpaste, gum, cigarettes, alcohol, meal including fish, spicy food, medication.

1.3. Fasting status at sample collection:

1.3.1. ***Standard collection:*** Collect sample after a fast of at least 6 hours.

1.3.2. ***Required minimum:*** Collect sample at least 1 hour after brushing teeth, at least 1 hour after eating a meal, at least 12 hours after last alcohol consumption, and at least 20 minutes after consuming acidic food (e.g., citrus fruits) or high sugar food.

1.4. Rinse mouth before collection of the sample.

1.5. Sample collection method:

1.5.1. ***Standard collection:*** Ask patient to drool into a sterile specimen container without the action of spitting. Saliva production can be enhanced by showing the patient mouth watering images such as lemons (no salivary stimulants). If collecting sample for DNA (e.g., Oragene®) follow the manufacturer’s protocol for collection.

1.5.2. ***Required minimum:*** Ask patient spit or drool into a sterile specimen container. Saliva production can be enhanced by showing the patient mouth watering images such as lemons (no salivary stimulants). Record the collection technique.

1.6. Amount of sample collection:

1.6.1. ***Standard collection:*** Obtain 2ml of saliva apart from foam/bubbles.

1.6.2. ***Required minimum:*** Obtain at least 1ml of saliva apart from foam/bubbles. If many bubbles form during collection, the participant can cap the container and gently tap the collection container on a hard surface.

1.7. Labelling of sample collection tubes:

1.7.1. ***Standard collection:*** Label the sample with a 2D barcode in addition to human-readable, showing the unique identifier of the patient and sample identifier. Record on the log sheet the date and time of sample collection (Date: __/__/__ and __:__am/pm).

1.7.2. ***Required minimum:*** Label the sample with the unique identifier of the patient and sample identifier. Record on the log sheet the date and time of sample collection (Date: __/__/__ and __:__am/pm).

**2. Sample processing in the laboratory, labelling aliquots and storage**

2.1. Place samples on wet ice or in refrigerator if there will be more than 1 hour before processing. If the time to processing is less than 1 hour, samples can be kept at room temperature.

2.2. Record on the log sheet the time of the sample processing started in the laboratory.

2.3. Pipette an appropriate amount of unprocessed saliva into a screw top vial with gasket closure and put on wet ice.

2.4. Preparation of sample aliquot tubes:

2.4.1. ***Standard collection:*** Label the aliquot vials with the participant ID number followed by a unique aliquot ID number. For example: ENDO-123456-U654321-S-01: Center identifier (ENDO), participant ID (123456), unique aliquot vial ID (U654321), sample type (S for saliva) and aliquot number (01). Also, include date of sample creation on the label to be able to distinguish samples from the same participant collected at different time points. Further, include the above information in human readable format and in a 2D barcode on the label.

2.4.2. ***Required minimum:*** Label the aliquot vials with the participant ID followed by the sample aliquot number. For example: ENDO-123456-S-01: Center identifier (ENDO), participant ID (123456), type of sample (S for saliva), aliquot number (01). Also, include date of sample creation on the label to be able to distinguish samples from the same participant collected at different time points.

2.5. Sample storage in freezers:

2.5.1. ***Standard collection:*** Store the unprocessed saliva aliquots in liquid nitrogen (LN_2_) freezers, which have less temperature fluctuations.

2.5.2. ***Required minimum:*** Store the unprocessed saliva aliquots in a freezer of -80°C or lower.

2.6. Centrifuge the remaining saliva sample in the collection tube at 1000g for two minutes at 4°C.

2.7. Aliquot appropriate amount of supernatant into an appropriate sized screw top vial with gasket closure. Label the aliquots as in 2.4. and store the processed saliva aliquots as in 2.5.

2.8. If interested in RNA extraction: Aliquot from the remaining supernatant in the collection tube after centrifugation to a different aliquot vial, which contains an RNA stabilizer [commercially available products: Allprotect Tissue Reagent® (Qiagen); DNA / RNA Shield™ (Zymoresearch); ProtectRNA™ (Sigma-Aldrich); RiboLock™ (Thermoscientific); RNAlater® (Qiagen); Ambion® [[RNAsecure™ Reagent](http://products.invitrogen.com/ivgn/product/AM7006) (Life-technologies); SUPERase•In™ (Life-technologies); PAXgene Tissue Containers (Qiagen)]](http://products.invitrogen.com/ivgn/product/AM2694). Label the aliquots as in 2.4. and store the processed RNA saliva aliquots as in 2.5.

2.9. Duration until sample aliquots are put into freezers for storage:

2.9.1. ***Standard collection:*** Samples should be processed and stored into freezers within maximum of 4 hours and time should be recorded on the log sheet. Also record the type, number and volume of aliquots prepared.

2.9.2. ***Required minimum:*** Record on the log sheet, the time of the sample processing completion/ time put into the freezer and type, number and volume of aliquots prepared.

2.10. Record any variations or deviations from the SOP, problems, or issues.

2.11. Record the location of each sample in the freezer including freezer number, rack, box, and position in the box along with all other sample attributes in a database. If possible, avoid using a spreadsheet format, but preferably use a relational database.

**3. Freezer check**

3.1.2. ***Standard collection:*** Split aliquots from the same sample type and individual between freezers in case of a freezer breaking down. Check freezers bi-weekly and keep a written-log of checks. Have alarm systems setup on all freezers in addition to human bi-weekly checks.

3.1.1. ***Required minimum:*** Manually check freezers bi-weekly and keep a written-log of checks.

**4. Data recording check list**

4.1. Record protocol, specifying which steps are adhered to (standard or minimum).

4.2. Record the time since the study participant ate or drank anything except plain water (Fasted since: __:__pm/am). Is it at least 6 hours? Yes/No.

4.3. Record whether it has been less than, (1) 1 hour after brushing teeth, (2) 1 hour after eating a meal, (3) 12 hours after last alcohol consumption, (4) 20 minutes after consuming acidic foods (e.g., citrus fruits) or high sugar foods.

4.4. For each sample, record:

4.4.1. Date and time of saliva collection (Date: __/__/__ and __:__am/pm).

4.4.2. Start time of sample processing in the laboratory (__:__am/pm).

4.4.3. Type, number and volume of aliquots prepared.

4.4.4. Date and time aliquots stored into freezers (Date: __/__/__ and __:__am/pm).

4.4.5. Any variations or deviations from the SOP, problems, or issues.

4.5. In the long-term, record:

4.5.1. Any freeze-thaw that occurs with a sample for any reason.

4.5.2. Any change of location of a sample, including sending a sample out to an assay lab for processing.

4.5.3. Any new samples created from the original aliquots (i.e., a sub-aliquot) in the same manner as described above.

4.6. Keep a bi-weekly log of freezer checks.
