# Supplementary material for: World Endometriosis Research Foundation Endometriosis Phenome and Biobanking Harmonization Project: III. Fluid biospecimen collection, processing, and storage in endometriosis research
Source: Fertil Steril. 2014 Nov;102(5):1233–43. doi: 10.1016/j.fertnstert.2014.07.1208 (PMC4230639; doi:10.1016/j.fertnstert.2014.07.1208)
Supplement: Supplemental Appendix 4 [file mmc10.docx]

**Supplemental Appendix IV:**

**Detailed standard operating procedure for the collection, processing and storage of endometrial fluid specimens**

**NOTES**

- This SOP does not cover safety procedures for the collection and processing of these samples and personnel must follow institutional biosafety guidelines**.**
- For a summary version of this protocol with side-by-side standard vs. minimal protocol step comparisons, please see Supplemental Table 4.
- As this protocol applies to different processing and storage methods, keep a copy of the exact step-by-step protocol used in your lab.

***Processing and storage materials***

1. Biospecimen form (Supplemental Appendix VII);
2. Log sheet to record sample-related data;
3. Embryo-transfer catheter and a 20ml syringe
4. Normal saline solution
5. Liquid nitrogen or dry ice
6. Crushed ice if a delay is anticipated
7. Transfer pipette
8. Volume adjustable pipette
9. Centrifuge
10. Labels suitable for long-term freezer storage, and IDs printed using 2D barcoding
11. Aliquot vials with screw top gasket closure
12. Freezers: -80C or liquid nitrogen (LN_2_)

**1. Endometrial fluid collection**

1.1. Time of sample collection in the clinic:

1.1.1. ***Standard collection:*** The sample is collected before any pre-medication/anesthesia administration.

1.1.2. ***Required minimum:*** Record whether sample is collected 1) prior to pre-medication, 2) after pre-medication but before anesthesia, or 3) after anesthesia.

1.2. Sample collection is performed by either (1) Aspiration method: Embryo-transfer catheter connected to a 20ml syringe to apply vacuum. (2) Lavage method: Slowly infuse and withdraw 4ml of saline solution into the uterine cavity. This uterine lavage fluid (ULF) can be processed as endometrial fluid, but the supernatant from ULF should be regarded with caution (see main text for discussion).

1.3. Record the time, date, volume and method of sample collection.

1.4. If the sample is collected using the embryo-transfer catheter, cut the tip of the catheter, insert into an aliquot tube and snap freeze immediately in liquid nitrogen.

1.5. If the lavage method is used Sample treatment until transported to the lab:

1.5.1. ***Standard collection:*** Transfer the sample into a screw top vial. If the amount of sample is small (not enough for centrifugation), put the sample immediately into liquid nitrogen or dry ice. If the amount of sample is enough for centrifugation put the sample on wet ice and transfer to the laboratory within 30 minutes.

1.5.2. ***Required minimum:*** Transfer the sample into a screw top vial. If the amount of sample is small (not enough for centrifugation), put the sample immediately into liquid nitrogen or dry ice. If the amount of sample is enough for centrifugation put the sample on wet ice and transfer to the laboratory as soon as possible.

**2. Sample processing in the laboratory**

2.1. Record on the log sheet the start time of sample processing in the laboratory.

2.2. Record colour, clarity and volume of the sample.

2.3. Centrifugation of samples:

2.3.1. ***Standard collection:*** Centrifuge samples for 5 minutes at 900g at 4°C. Keep a record of the standard time and g in your adapted SOP.

2.3.2. ***Required minimum:*** Centrifuge samples for 5 minutes at 900g at room temperature. Keep a record of the standard time and g in your adapted SOP.

2.4. Transfer the supernatant to appropriate sized aliquot vial with screw top gasket closure (not ‘push fit top’ as they are not airtight) and fill as close to full as possible to minimise surface area. If ULF method is used for collection, be cautious in downstream analyses of the supernatant (See main text for discussion).

2.5. Transfer the pellet to appropriate sized aliquot vial with screw top gasket closure (not ‘push fit top’ as they are not airtight).

**3. Labelling aliquots and storage**

3.1. Use special labels and ink that do not disintegrate when stored in very low temperature freezers (i.e. Do not use laser printers or most ink-printers as they disintegrate when frozen).

3.2. Preparation of sample aliquot tubes:

3.2.1. ***Standard collection:*** Label the aliquot vials with the participant ID number followed by a unique aliquot ID number. For example: ENDO-123456-U654321-EF-01: Center identifier (ENDO), participant ID (123456), unique aliquot vial ID (U654321), sample type (EF for endometrial fluid) and aliquot number (01). Also, include date of sample creation on the label to be able to distinguish samples from the same participant collected at different time points. Further, include the above information in human readable format and in a 2D barcode on the label.

3.2.2. ***Required minimum:*** Label the aliquot vials with the participant ID followed by the sample aliquot number. For example: ENDO-123456-EF-01: Center identifier (ENDO), participant ID (123456), type of sample (EF for endometrial fluid), aliquot number (01). Also, include date of sample creation on the label to be able to distinguish samples from the same participant collected at different time points.

3.3. Record the time of the sample processing completion/ time put into the freezer. Also record the type, number and volume of aliquots prepared.

3.4. If storing unprocessed small amount of sample in freezers:

3.4.1. ***Standard collection:*** Transfer the small, unprocessed sample from dry ice or liquid nitrogen to liquid nitrogen (LN_2_) freezers for long-term storage.

3.4.2. ***Required minimum:*** Transfer the small unprocessed sample from dry ice or liquid nitrogen to -80°C or lower freezers for long term storage.

3.5. If storing processed sample separated into supernatant and pellet in freezers:

3.5.1. ***Standard collection:*** Store fluid aliquots in liquid nitrogen (LN_2_) freezers, which have less temperature fluctuations.

3.5.2. ***Required minimum:*** Store fluid aliquots at -80°C or lower freezers for long term storage.

3.6. Record any variations or deviations from the SOP, problems, or issues.

3.7. Record the location of each sample in the freezer including freezer number, rack, box, and position in the box along with all other sample attributes in a database. If possible avoid using a spreadsheet format, but preferably use a relational database.

**4. Freezer check**

4.1.1. ***Standard collection:*** Split aliquots from the same sample type and individual between freezers in case of a freezer breaking down. Check freezers bi-weekly and keep a written-log of checks. Have alarm systems setup on all freezers in addition to human bi-weekly checks.

4.1.2. ***Required minimum:*** Manually check freezers bi-weekly and keep a written-log of checks.

**5. Data recording check list**

5.1. Record protocol, specifying which steps are adhered to (standard or minimum).

5.2. For each sample, record:

5.2.1. Date and time of fluid collection (Date: __/__/__ and __:__am/pm).

5.2.2. Start time of sample processing in the laboratory (__:__am/pm).

5.2.3. Type, number and volume of aliquots prepared.

5.2.4. Date and time aliquots stored into freezers (Date: __/__/__ and __:__am/pm).

5.2.5. Any variations or deviations from the SOP, problems, or issues.

5.3. In the long-term, record:

5.3.1. Any freeze-thaw that occurs with a sample for any reason.

5.3.2. Any change of location of a sample, including sending a sample out to an assay lab for processing.

5.3.3. Any new samples created from the original aliquots (i.e., a sub-aliquot) in the same manner as described above.

5.4. Keep a bi-weekly log of freezer checks.
