# Supplementary material for: World Endometriosis Research Foundation Endometriosis Phenome and Biobanking Harmonization Project: III. Fluid biospecimen collection, processing, and storage in endometriosis research
Source: Fertil Steril. 2014 Nov;102(5):1233–43. doi: 10.1016/j.fertnstert.2014.07.1208 (PMC4230639; doi:10.1016/j.fertnstert.2014.07.1208)
Supplement: Supplemental Appendix 6 [file mmc12.docx]

**Supplemental Appendix VI:**

**Detailed standard operating procedure for the collection, processing and storage of menstrual effluent (blood) specimens**

**NOTES**

- This SOP does not cover safety procedures for the collection and processing of these samples and personnel must follow institutional biosafety guidelines**.**
- For a summary version of this protocol with side-by-side standard vs. minimal protocol step comparisons, please see Supplemental Table 6.
- As this protocol applies to different processing and storage methods, keep a copy of the exact step-by-step protocol used in your lab.

***Processing and storage materials***

1. Biospecimen form (Supplemental Appendix VII)
2. Log sheet to record sample-related data
3. A diaphragm or a mixing cannula
4. Sterile closed container for transfer of the sample to the lab
5. Crushed ice if a delay is anticipated
6. Transfer pipette
7. Volume adjustable pipette
8. Centrifuge
9. EDTA/heparin tubes if plasma is going to be collected from the sample
10. Labels suitable for long-term freezer storage, and IDs printed using 2D barcoding
11. Aliquot vials with screw top gasket closure
12. Freezers: -80C or liquid nitrogen (LN_2_)

**1. Menstrual effluent (blood) collection**

1.1. Collect menstrual effluent sample with a diaphragm or mixing cannula.

1.2. Record on the log sheet the day of the menstrual cycle of the sample collection (Day __) and the date and time of menstrual effluent collection (Date: __/__/__ and __:__am/pm).

1.3. Labelling of sample collection container:

1.3.1. ***Standard collection:*** Once the collection container is collected from the patient label the sample with a 2D barcode in addition to human-readable the unique identifier of the patient and sample identifier.

1.3.2. ***Required minimum:*** Once the collection container is collected from the patient, label the sample with the unique identifier of the patient and sample identifier.

1.4. Sample treatment until transported to the lab:

1.4.1. ***Standard collection:*** The sample is put in a closed container and transferred and processed in the laboratory within 1 hour on wet ice.

1.4.2. ***Required minimum:*** The sample is put in a closed container and transferred and processed in the laboratory within 1 hour at room temperature.

**2. Sample processing in the laboratory, labelling aliquots and storage**

2.1. Record start time of sample processing in the laboratory. Sample should be processed within a maximum of 1 hour.

2.2. In the laboratory, if an unprocessed sample is required for the study, transfer sample into appropriate sized aliquot vial with screw top gasket closure.

2.3. Preparation of sample aliquot tubes:

2.3.1. ***Standard collection:*** Label the aliquot vials with the participant ID number followed by a unique aliquot ID number. For example: ENDO-123456-U654321-ME: Center identifier (ENDO), participant ID (123456), unique aliquot vial ID (U654321), sample type (ME for menstrual effluent) and aliquot number (01). Also, include date of sample creation on the label to be able to distinguish samples from the same participant collected at different time points. Further, include the above information in human readable format and in a 2D barcode on the label.

2.3.2. ***Required minimum:*** Label the aliquot vials with the participant ID number followed by a unique aliquot ID number. For example: ENDO-123456-U654321-ME: Center identifier (ENDO), participant ID (123456), unique aliquot vial ID (U654321), sample type (ME for menstrual effluent) and aliquot number (01). Also, include date of sample creation on the label to be able to distinguish samples from the same participant collected at different time points.

2.4. Sample storage in freezers:

2.4.1. ***Standard collection:*** Store the unprocessed menstrual effluent aliquots in liquid nitrogen (LN_2_) freezers, which have less temperature fluctuations.

2.4.2. ***Required minimum:*** Store the unprocessed menstrual effluent aliquots at -80°C or lower freezers.

2.5. Samples should be stored into freezers within maximum of 1 hour and time should be recorded on the log sheet. Also record the type, number and volume of aliquots prepared.

2.6. If a plasma sample is required for the study, the remaining sample from the collection container is transferred into EDTA/heparin tubes and placed on wet ice. Record which tubes is used.

2.7. Centrifuge sample for 10 minutes at 2500 x g at 4°C.

2.8. Aspirate plasma using an appropriate transfer pipette (or ideally a volume-adjustable pipette) into appropriate sized aliquot vials. Label the aliquots as in 2.3, store the processed plasma aliquots as in 2.4 and keep the record of time and type of aliquots prepared as in 2.5.

2.9. Record any variations or deviations from the SOP, problems, or issues.

2.10. Record the location of each sample in the freezer including freezer number, rack, box, and position in the box along with all other sample attributes in a database. If possible avoid using a spreadsheet format, but preferably use a relational database.

**3. Freezer check**

3.1.2. ***Standard collection:*** Split aliquots from the same sample type and individual between freezers in case of a freezer breaking down. Check freezers bi-weekly and keep a written-log of checks. Have alarm systems setup on all freezers in addition to human bi-weekly checks.

3.1.1. ***Required minimum:*** Manually check freezers bi-weekly and keep a written-log of checks.

**4. Data recording Check list**

4.1. Record protocol, specifying which steps are adhered to (standard or minimum).

4.2. Record date and time of menstrual effluent collection (Date: __/__/__ and __:__am/pm).

4.3. Record cycle day on the menstrual effluent collection (Day __).

4.4. For each sample, record:

4.4.1. Date and time of fluid collection (Date: __/__/__ and __:__am/pm).

4.4.2. Start time of sample processing in the laboratory (__:__am/pm).

4.4.3. Type, number and volume of aliquots prepared.

4.4.4. Date and time aliquots stored into freezers (Date: __/__/__ and __:__am/pm).

4.4.5. Any variations or deviations from the SOP, problems, or issues.

4.5. In the long-term, record:

4.5.1. Any freeze-thaw that occurs with a sample for any reason.

4.5.2. Any change of location of a sample, including sending a sample out to an assay lab for processing.

4.5.3. Any new samples created from the original aliquots (i.e., a sub-aliquot) in the same manner as described above.

4.6. Keep a bi-weekly log of freezer checks.
