# Supplementary material for: World Endometriosis Research Foundation Endometriosis Phenome and Biobanking Harmonization Project: III. Fluid biospecimen collection, processing, and storage in endometriosis research
Source: Fertil Steril. 2014 Nov;102(5):1233–43. doi: 10.1016/j.fertnstert.2014.07.1208 (PMC4230639; doi:10.1016/j.fertnstert.2014.07.1208)
Supplement: Supplemental Appendix 7 [file mmc13.docx]

**Supplemental Appendix VII:**

**EPHect Biospecimen Collection Form** (to be completed by research nurse)

Date and time sample collected: (DD/MM/YYYY) ___ / ___ / _______ Time: ____:____ AM/PM

What was the first day of your last menstrual period? (DD/MM/YYYY) ___ / ___ / _______

Are your periods regular? (Predictable within one week)

🞏 Yes 🞏 No

Specify range of days : [regular range: 21-35days]

If you have not had a menstrual period in the past 90 days, please tell us why:

##### 🞎 Taking hormones continuously (*e.g. the Pill, injections, Mirena, HRT*)

##### 🞎 Pregnant

##### 🞎 Breastfeeding

##### 🞎 Unsure

##### 🞎 Other (*Please describe*) ________________________________________________________

Are you currently having a menstrual period/vaginal bleeding *(including spotting for which you only need a panty liner)*?

🞎 No

🞎 Yes, menstrual period

🞎 Yes, irregular bleeding/spotting

Do you currently have a coil [IUD] in place?

🞎 No

🞎 Yes → If yes, what kind of IUD? 🞎 Progesterone containing IUD (Mirena)

🞎 Other coil/intrauterine device

When was the last time you had something to eat?

____ : ____ am/pm 🞎 Today 🞎 Yesterday

When was the last time you had something to drink (other than plain water) and what did you drink?

____ : ____ am/pm 🞎 Today 🞎 Yesterday

____________________________________________________________________________

**Clinical Measurements:**

Height: ______ in cm, or ______ in inches

Weight: ______ in kg, or ______ in pounds

Hip circumference: ______ in cm, or ______ in inches

Waist circumference: ______ in cm, or ______ in inches

*See NHANES III video guidelines on how to take measurements (also included as text in the Supplementary Appendix VIII):* https://www.youtube.com/watch?v=KacU_TW50Zo

**If saliva samples are being collected:**

Please indicate whether or not you have used the following in the last 24 hours and what time you used each item.

| Toothpaste | 🞎 No 🞎 Yes → | ___ : ___ AM/PM 🞎 Today 🞎 Yesterday |
| --- | --- | --- |
| Gum | 🞎 No 🞎 Yes → | ___ : ___ AM/PM 🞎 Today 🞎 Yesterday |
| Cigarettes | 🞎 No 🞎 Yes → | ___ : ___ AM/PM 🞎 Today 🞎 Yesterday |
| Alcohol | 🞎 No 🞎 Yes → | ___ : ___ AM/PM 🞎 Today 🞎 Yesterday |

In the past 24 hours have you eaten:

Spicy food? 🞎 No 🞎 Yes

Fish? 🞎 No 🞎 Yes

**If urine samples are being collected:**

When did you last urinate (prior to providing the sample)?

____ : ____ am/pm 🞎 Today 🞎 Yesterday

What time was the urine sample produced?

____ : ____ am/pm

Is this urine sample your first morning void?

🞎 No

🞎 Yes → If yes, did you get up during the night to urinate? 🞎 No 🞎 Yes

In collecting this sample, did you follow a clean catch protocol?

🞎 No

🞎 Yes

**If undergoing an operation:**

Was any pre-med taken before blood, urine, saliva, endometrial fluid and eutopic endometrium/ myometrium collection? (NB. EPHect recommends sample taking prior to pre-med administration)

🞏 No

🞏 Yes

If yes, tick which samples were taken after pre-med administration:

🞏Blood 🞏Urine 🞏Saliva 🞏Endometrial fluid

🞏Eutopic endometrium/ myometrium

Time pre-med was administered: ____am/pm

Please specify the type of pre-med was administered _____________________________

Was anaesthetic administered before blood, endometrial fluid and eutopic endometrium collection?

🞏 No

🞏 Yes

If yes, tick which samples were taken after anaesthesia administration:

🞏 Blood 🞏 Endometrial fluid 🞏 Eutopic endometrium

If yes, time anaesthetic was administered: ____am/pm

Please specify the type of pre-med was administered: __________________________

**Method(s) of excision:**

Ectopic endometrium

🞏 Electrosurgery

🞏 Harmonic scalpel

🞏 Laser [CO_2_, NdYag and others]

🞏 Cold scissors/scapels

Eutopic endometrium

🞏 Endometrial sampling device

🞏 Curettage with cervical dilation

🞏 Brushing

Myometrium

🞏 Laser [CO_2_, NdYag and others]

🞏 Electrosurgery

🞏 Cold scissors/scapels

🞏 TruCut biopsy

Peritoneum

🞏 laser [CO_2_, NdYag and others]

🞏 Electrosurgery

🞏 Ultrasound energy

🞏 Harmonic scalpel

🞏 Cold scissors/scapels

🞏 Brushing

**Method(s) of collection:**

Peritoneal fluid

🞏 No lavage. Amount collected ___ml

🞏 Lavage method with 10ml sterile saline solution. Amount of peritoneal lavage fluid (PLF) ___ml

Endometrial fluid

🞏 No lavage. Amount collected ___ml

🞏 Lavage method with 4ml sterile saline solution. Amount of uterine lavage fluid (ULF) ___ml

**Use of prescription drugs, non-prescription drugs, vitamins or supplements** **in the past 30 days.**

| **Type of drug** | **Have you ever taken this drug every day for over a month?** | **At what age did you first take this drug every day for over a month?** | **In total, how many years you have taken this drug? Please estimate, and enter “0 total years” if less than 1 year.** | **Are you currently taking this drug every day?** | **Please write down the specific name of the drug you have used most recently if known:** |
| --- | --- | --- | --- | --- | --- |
| **PRESCRIPTION DRUGS** | ✓ *if yes* | *Age 1^st^* | *Years taken:* | ✓ *if yes* | *Name of drug:* |
| a. Hormonal medications | 🞎 | __ __ | __ __ | 🞎 |  |
| Birth control pill | 🞎 | __ __ | __ __ | 🞎 |  |
| Progestin injection/shot | 🞎 | __ __ | __ __ | 🞎 |  |
| Transdermal patch/dot | 🞎 | __ __ | __ __ | 🞎 |  |
| Vaginal ring | 🞎 | __ __ | __ __ | 🞎 |  |
| Progesterone containing coil/IUD | 🞎 | __ __ | __ __ | 🞎 |  |
| Hormonal implant | 🞎 | __ __ | __ __ | 🞎 |  |
| Oral progestins to regulate cycle | 🞎 | __ __ | __ __ | 🞎 |  |
| GnRH agonist infection/shot | 🞎 | __ __ | __ __ | 🞎 |  |
| Norethindrone acetate | 🞎 | __ __ | __ __ | 🞎 |  |
| Danazol | 🞎 | __ __ | __ __ | 🞎 |  |
| Hormone replacement therapy (HRT) | 🞎 | __ __ | __ __ | 🞎 |  |
| Other: ……………………………………………… | 🞎 | __ __ | __ __ | 🞎 |  |
| b. Pain medications | 🞎 | __ __ | __ __ | 🞎 |  |
| Paracetamol/acetaminophen | 🞎 | __ __ | __ __ | 🞎 |  |
| Aspirin | 🞎 | __ __ | __ __ | 🞎 |  |
| Ibuprofen | 🞎 | __ __ | __ __ | 🞎 |  |
| COX-2 inhibitors (e.g. celebrex, vioxx) | 🞎 | __ __ | __ __ | 🞎 |  |
| Other anti-inflammatory analgesics  (e.g. naproxen, mefanamic acid, aleve,  naprosyn, relafen, keoprofen, anaprox) | 🞎 | __ __ | __ __ | 🞎 |  |
| Narcotic analgesics (e.g. hydrocodone+  paracetamol, codeine, morphine) | 🞎 | __ __ | __ __ | 🞎 |  |
| Muscle relaxants (e.g. diazepam/  temazepam, buscopan) | 🞎 | __ __ | __ __ | 🞎 |  |
| Other: ……………………………………………… | 🞎 | __ __ | __ __ | 🞎 |  |
| c. Diuretic (water pill) | 🞎 | __ __ | __ __ | 🞎 |  |
| d. Diabetic tablets | 🞎 | __ __ | __ __ | 🞎 |  |
| e. Insulin | 🞎 | __ __ | __ __ | 🞎 |  |
| f. Thyroid drugs | 🞎 | __ __ | __ __ | 🞎 |  |
| g. Drugs for epilepsy | 🞎 | __ __ | __ __ | 🞎 |  |
| h. Sleeping tablets / tranquilisers | 🞎 | __ __ | __ __ | 🞎 |  |
| i. Anti-depressants | 🞎 | __ __ | __ __ | 🞎 |  |
| j. Other drugs to treat mental illness | 🞎 | __ __ | __ __ | 🞎 |  |
| k. Drugs for osteoporosis (“brittle bones”) | 🞎 | __ __ | __ __ | 🞎 |  |
| l. Drugs for rheumatoid arthritis | 🞎 | __ __ | __ __ | 🞎 |  |
| m. Antibiotics for a month or more | 🞎 | __ __ | __ __ | 🞎 |  |
| n. Antacids | 🞎 | __ __ | __ __ | 🞎 |  |
| o. Drugs for stomach ulcer / gastritis | 🞎 | __ __ | __ __ | 🞎 |  |
| p. Drugs for high cholesterol | 🞎 | __ __ | __ __ | 🞎 |  |
| q. Drugs for allergies (antihistamines) | 🞎 | __ __ | __ __ | 🞎 |  |
| r. Steroids (oral, inhaled, or nasal) | 🞎 | __ __ | __ __ | 🞎 |  |
| s. Chemotherapy for cancer | 🞎 | __ __ | __ __ | 🞎 |  |
| t. Tamoxifen for cancer | 🞎 | __ __ | __ __ | 🞎 |  |
| u. Blood pressure drugs | 🞎 | __ __ | __ __ | 🞎 |  |
| v. Drugs for angina (chest pain) | 🞎 | __ __ | __ __ | 🞎 |  |
| w. Other drugs for a heart condition | 🞎 | __ __ | __ __ | 🞎 |  |
| x. Inhaler for asthma | 🞎 | __ __ | __ __ | 🞎 |  |
| y. Warfarin / heparin to thin blood | 🞎 | __ __ | __ __ | 🞎 |  |
| z. Migraine tablets/injections | 🞎 | __ __ | __ __ | 🞎 |  |
| Other 1: ……………………………. | 🞎 | __ __ | __ __ | 🞎 |  |
| Other 2: …………………………….. | 🞎 | __ __ | __ __ | 🞎 |  |
| Other 3: …………………………….. | 🞎 | __ __ | __ __ | 🞎 |  |
| Other 4: …………………………….. | 🞎 | __ __ | __ __ | 🞎 |  |
| Other 5: …………………………….. | 🞎 | __ __ | __ __ | 🞎 |  |

| **Type of drug** | **Have you taken this drug in the past 30 days?** | **Have you taken this drug in the past 48 hours**  **(2 days)?** | **In the past 30 days, on how many days have you taken this drug?** | **Please write down the specific name of the drug**  **if known:** |
| --- | --- | --- | --- | --- |
| **NON-PRESCRIPTION DRUGS** | *✓ if yes* | *✓ if yes* | *Number of days:* | *Name of drug:* |
| a. Aspirin | 🞎 | 🞎 | __ __ days |  |
| b. Paracetamol | 🞎 | 🞎 | __ __ days |  |
| c. Ibuprofen | 🞎 | 🞎 | __ __ days |  |
| d. Other anti-inflammatory analgesics (e.g. naproxen) | 🞎 | 🞎 | __ __ days |  |
| e. Herbal pain medication: ……………….. | 🞎 | 🞎 | __ __ days |  |
| f. Other pain medication: ………………….. |  |  |  |  |
| g. Migraine tablets | 🞎 | 🞎 | __ __ days |  |
| h. Antihistamine for allergies | 🞎 | 🞎 | __ __ days |  |
| i. Cold medicine / lemsip | 🞎 | 🞎 | __ __ days |  |
| j. Decongestant | 🞎 | 🞎 | __ __ days |  |
| k. Cough syrup | 🞎 | 🞎 | __ __ days |  |
| l. Antacids | 🞎 | 🞎 | __ __ days |  |
| m. Sleeping tablets | 🞎 | 🞎 | __ __ days |  |
| n. Eye drops | 🞎 | 🞎 | __ __ days |  |
| o. Vaginal thrush treatments (cream or tablets) | 🞎 | 🞎 | __ __ days |  |
| p. Cystitis treatments / cymalon | 🞎 | 🞎 | __ __ days |  |
| q. Mouth ulcer treatments | 🞎 | 🞎 | __ __ days |  |
| r. Nicotine replacement treatments | 🞎 | 🞎 | __ __ days |  |
| Other 1:......................................... | 🞎 | 🞎 | __ __ days |  |
| Other 2:......................................... | 🞎 | 🞎 | __ __ days |  |
| Other 3:......................................... | 🞎 | 🞎 | __ __ days |  |
| Other 4:......................................... | 🞎 | 🞎 | __ __ days |  |
| Other 5:......................................... | 🞎 | 🞎 | __ __ days |  |
|  |  |  |  |  |
| **VITAMINS & SUPPLEMENTS** |  |  |  |  |
| #1: ................................................. | 🞎 | 🞎 | __ __ days |  |
| #2: ................................................. | 🞎 | 🞎 | __ __ days |  |
| #3: ................................................. | 🞎 | 🞎 | __ __ days |  |
| #4: ................................................. | 🞎 | 🞎 | __ __ days |  |
| #5: ................................................. | 🞎 | 🞎 | __ __ days |  |
| #6: ................................................. | 🞎 | 🞎 | __ __ days |  |
| #7: ................................................. | 🞎 | 🞎 | __ __ days |  |
| #8: ................................................. | 🞎 | 🞎 | __ __ days |  |
| #9: ............................................... | 🞎 | 🞎 | __ __ days |  |
| #10: ............................................... | 🞎 | 🞎 | __ __ days |  |
