# Supplementary material for: World Endometriosis Research Foundation Endometriosis Phenome and Biobanking Harmonization Project: III. Fluid biospecimen collection, processing, and storage in endometriosis research
Source: Fertil Steril. 2014 Nov;102(5):1233–43. doi: 10.1016/j.fertnstert.2014.07.1208 (PMC4230639; doi:10.1016/j.fertnstert.2014.07.1208)
Supplement: Supplemental Appendix 8 [file mmc14.docx]

**Supplemental Appendix VIII:**

**Measurement of waist and hip circumference**

**I. Waist Circumference:** palpate the hip area for the right iliac crest and mark a horizontal line at the high point of the iliac crest and then crosses the line to indicate the midaxillary line of the body. The pants and underclothing of the subject should be lowered slightly to allow for the examiner to palpate directly on the hip area for the iliac crest. Place the measuring tape around the trunk in a horizontal plane at the marked level, make sure the tape is parallel to the floor and take the measurement.

**II. Hip circumference:** measure around the widest portion of the buttocks, with the tape parallel to the floor. Any extra thick clothing should be removed before taking the measurement.

For both measurements, the subject should stand straight with feet close together, arms at the side and body weight evenly distributed, and should wear little clothing. The subject should be relaxed, and the measurements should be taken at the end of a normal expiration to the nearest 0.1 cm. The measuring tape should be held snug but not tight.

Please see NHANES II video guidelines on how to take these measurements: https://www.youtube.com/watch?v=KacU_TW50Zo
